# Supplementary material for: Swab-Seq: A high-throughput platform for massively scaled up SARS-CoV-2 testing
Source: medRxiv. 2021 Mar 9:2020.08.04.20167874. Preprint. [Version 4] doi: 10.1101/2020.08.04.20167874 (PMC7480060; doi:10.1101/2020.08.04.20167874)
Supplement: Supplement 2020 [file 95337-2020.08.04.20167874-1.docx]

**Standard Operating Procedure**

**UCLA SwabSeq COVID-19 Test**

1. **PURPOSE**

Swab-seq technology is a unique approach to viral testing that eliminates many of the current bottlenecks that limit clinical testing and, importantly, can ***rapidly scale to 10,000 samples per day*** with simple automation and many of the machines and semi-automation that are standard in research laboratories. This technology holds immense promise for ***large-scale population screening for COVID19.*** The major limitations to wide-spread deployment of this technology is logistics regarding specimen collection, linking with symptom surveys, contact tracing efforts, and returning results. Swab-seq is:

- Simple (5 steps from sample to sequencing)
- Inexpensive (<$1/sample in consumable costs)
- Scalable (~10K samples/day, w/ automation, 100K/day or more depending on standard thermocycler capacity)
- Sensitive (LoD ~1-6 molecules/test) and quantitative (> 3-4 logs)


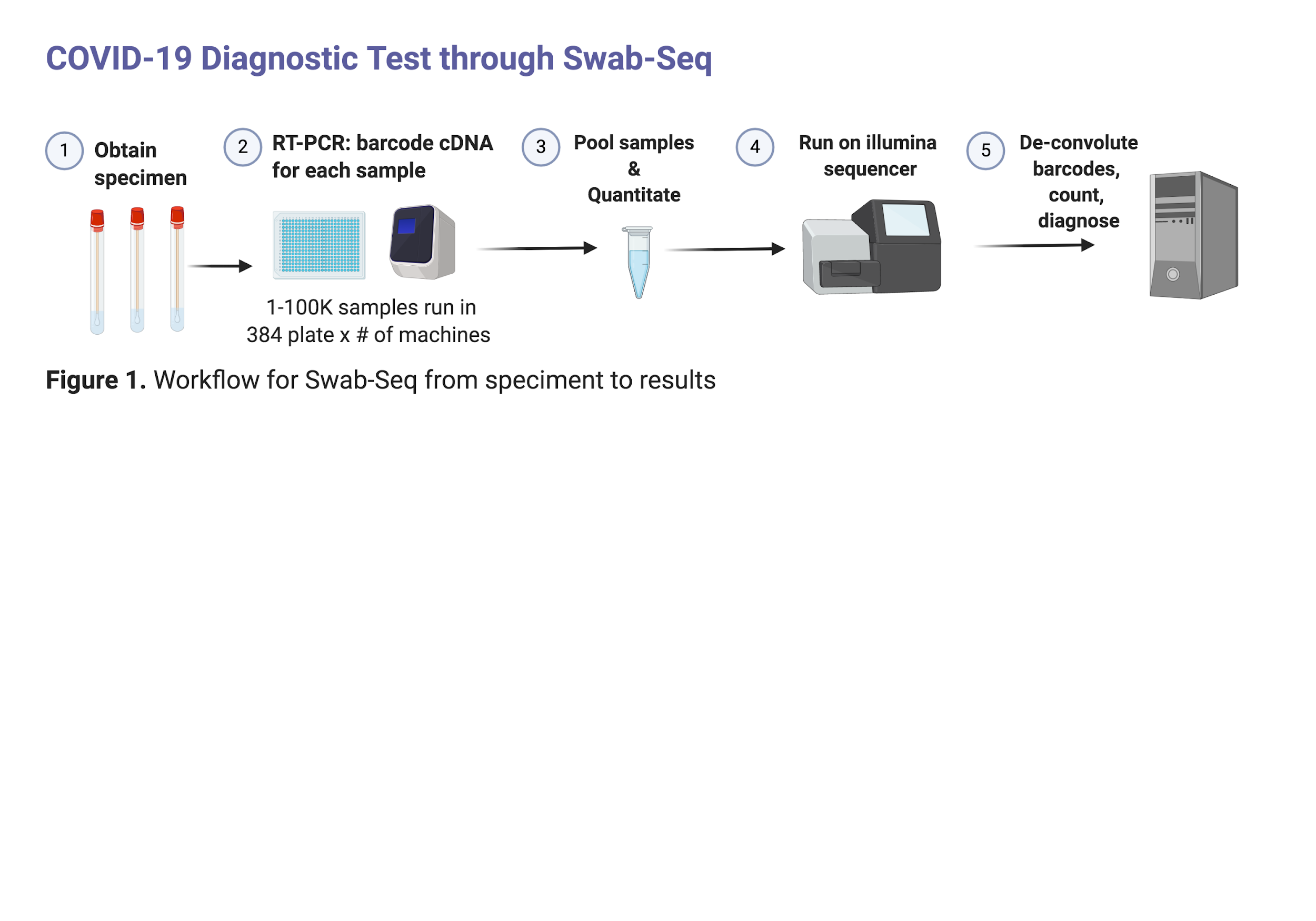


1. **SPECIMEN COLLECTION**

Please refer to separate SOP for Specimen Collection and Handling. This protocol is designed to work on upper respiratory tract specimens collected through approved protocols at UCLA Health.

1. **MATERIALS AND EQUIPMENT**

**Equipment**

Veriti 384-Well Thermal Cycler (Fisher Scientific, A41191) x 8

Plate Centrifuge (Eppendorf, 5920R)

Multichannel Pipet (Eppendorf, 12-channel, 10, 100, 1000)

BenchSmart96 20ul BST-96-20 (Rainin, 30296705)

BenchSmart96 200ul BST-96-200 (Rainin, 30296706)

Integra Viaflo 384-well Unit

(Base Unit 6031, 384 Channel Pipetting Head (0.5-12.5 μl) 6131, 3-position state, 6230)

MiSeq (Illumina)

Tape Station 4150 (Agilent)

QUBIT 4 NGS (Fisher, Q33228)

**Reagents and Materials Needed**

General

Pipette Tips LQR LTS 20μL FL 960/10 (Rainin, 17014400 for Bench Smart)

12.5 μl GRIPTIP Sterile, Filter, LONG, 5 XYZ Racks of 384 Tips, Low Retention (Integra, 6505)

384-WELL HARDSHELL PLATE CLEAR (#20 PCS PK 4483285 Fisher)

TaqPath™ 1-Step RT-qPCR Master Mix (Fisher Scientific, A15300)

TE Buffer, Tris-EDTA, 1X Solution, pH 7.4 (Fisher BP24761)

10X TBE Buffer, 1 M Tris, 0.9 M boric acid, and 0.01 M EDTA (Fisher 15581044)

Tween-20

EtOH

NUCLEASE-FREE WATER (Thermofisher, AM9937)

96-well plates (qRT-PCR)

NaOH

Synthetic S2 Spike Construction

# HiScribe™ T7 High Yield RNA Synthesis Kit (New England Biolabs, E2040S)

# DNAse, RNAse Free (NEB, M0303S)

# RNA Clean & Concentrator-5 (Zymo, [R1013](https://www.zymoresearch.com/collections/rna-clean-concentrator-kits-rcc/products/rna-clean-concentrator-5))

Post-PCR Pooling and Purification

DynaMag-2 Magnet (Thermofisher Scientific, 12321D)

Ampure XP Beads (Fisher Scientific, A63880)

1.7 mL Eppendorf Lo-Bind Tubes (Fisher Scientific, 13-698-791)

150 mL reservoir, sterile, bulk, automation friendly, polystyrene (Integra, 6318)

Qubit™ RNA HS Assay Kit (ThermoFisher, Q32855)

Qubit™ Assay Tubes (Thermofisher Scientific, Q32856)

Qubit™ RNA BR Assay Kit (ThermoFisher, Q10211)

Sequencing Materials and Reagents

PhiX Control v3 (Illumina, FC-110-3001)

Illumina Free Adapter Blocking Reagent (48 reactions, # 20024145)

MiSeq Reagent Kit v3 (150-cycle, Catalog # MS-102-3001)

1. **PRIMERS**

Indexed Primers

Custom Primer sets were designed to amplify the S2 gene in the SARS-CoV2 genome and the RPP30 gene in the human genome. The S2 gene amplification indicates the presence of SARS-CoV2 RNA genome within the specimen. The RPP30 gene demonstrates adequate sample collection. Each primer pair (i5 and i7, designated as F and R) has a 10 bp unique barcode for identifying a specific sample. The i5 barcode can be repeated over the plate and is sometimes referred to as the “plate barcode”. The combinatorial indexing strategy reduces the number of unique primers that need to be purchased. An alternative, but more expensive strategy, is to purchase unique-dual indices (UDI) which are designed to be a unique pair in each well.

**S2 and RPP30 Primers**, Custom Primers were obtained from:

<https://www.notion.so/Octant-SwabSeq-Testing-9eb80e793d7e46348038aa80a5a901fd>

Ordered from IDT

100nM, 200uM concentration, Standard Purification

**Final Concentration for Working Primers (10x):**

4 μM for S2

0.5 μM for RPP30

**Custom Sequencing Primers:**

Ordered from Integrated DNA Technologies

100nM synthesis scale, concentration is 100uM

| **S2_SARS-CoV-2** |  | **Tm** |
| --- | --- | --- |
| Read_1 | gctggtgctgcagcttattatgtgggt | 63 |
| i7_seq | agatgctgtagactgtgcacttgaccct | 63 |
| i5_seq | acccacataataagctgcagcaccagc | 63 |
|  |  |  |
| **RPP30** |  |  |
| Read_1 | gagcggctgtctccacaagtccg | 63.5 |
| i7_seq | acccgctcgcaggtccaaatct | 62.6 |
| i5_seq | cggacttgtggagacagccgctc | 63.5 |

**Table 1:** Primers used for specific amplification of S2 gene and human control RPP30.

1. **PRIMER STAMPING**

All plates will be pre-stamped with a mix of indexed primers at 10x concentration (4 μM for S2For and S2Rev; 0.5 μM for RPP30For and RPP30Rev). For a 20 μL reaction, we will place 2uL into each well of the primer plate.

1. Spin down master plates at 2000xg for 1 minute to ensure that all frozen ice is at the bottom of the well. This prevents cross contamination of indexed primers when removing the foil lid.
2. Set up Integra work station:
   1. Scan or write down Barcode Labs in the Primer Plate Notebook
   2. Label multiple -384-well primer plates with primer set name.
   3. Change the Setting on the Integra for multi-dispensing of 2uLs per plate x 5 plates
3. Remove seal from the master plates very carefully
4. Using the 384-well head for the Integra Viaflo, carefully pipet up primer plates and dispense 2uL into each plate.
5. Seal each plate, freeze in -20 until use.

**Repeat with each 384-well Primer Plate Set.**

1. **CONSTRUCTION OF SYNTHETIC S2 RNA SPIKE**

**Purpose:** We use a synthetic RNA spike-in as a control in each of our wells. The sequence is meant to mimic the actual SARS CoV-2 amplicon (same amplicon structure except for a 6 nt unique stretch to distinguish). The use of this synthetic spike has two important advantages.

1. This serves as an in-well positive control for the S2 primers. Even in a sample that has no SARS-CoV-2, we can observe amplification with primers, thereby ensuring that a negative result is due to lack of virus, and not due to technical issues with the primers.
2. A second benefit is that we can use the ratio between S2/S2 spike to demonstrate that small changes in experimental conditions such as differences in sample inhibitory properties, small differences in pipetting volumes, noise and amplification biases are not affecting our results.

**RT-PCR primers for spike constriction using *In vitro* Transcription (adding on T7 promoter)**

| S2_FP | TAATACGACTCACTATAGggctggtgctgcagcttattatgtgggtATAGAAcaacctaggacttttctattaa |
| --- | --- |
| S2_RP | aacgtacactttgtttctgagagagg |

**Table 2:** Primers to construct template for the S2 Synthetic Spike.

1. Perform RT-PCR in a 96-Well thermocycler using the primers above and gRNA of SARS-CoV-2.
2. Run on an Agarose gel and ensure specific products at ~130 bp
3. Purify DNA using Ampure Beads, use a ratio of 1.8 ratio of beads: sample volume.
4. Vortex and let sit for 5 minutes at room temperature.
5. Use magnet to collect beads for 1 minute.
6. Remove liquid and wash beads twice with 500 ul of freshly made 70% EtOH.
7. Elute in 100uL of 0.1X Qiagen EB buffer.
8. Use a magnet to collect beads for 1 minute. Transfer 90 uL to the new eppendorf tube.
9. Quantify samples using Qubit DNA BR Kit.

Store DNA overnight or at -20 for long term storage if not immediately proceeding to IVT reaction.

1. Thaw the necessary kit components from the NEB HiScribe Kit. Mix and pulse-spin in microfuge to collect solutions to the bottom of tubes. Keep on ice.
2. Prepare MasterMix for In Vitro Translation Reaction:
   1. If you are planning to run many reactions, it is convenient to prepare a master mix by combining equal volumes of the 10X reaction buffer and four ribonucleotide (NTP) solutions
   2. Using this mastermix (see below Table 1) we made 4 reactions.
   3. Split into 4 tubes with 20 μl each.
   4. Vortex, pulse spin, but at 37˚C overnight in the Thermocycler.

| **Component** | **per reaction** | **Property** | **4 reactions (μl)** |
| --- | --- | --- | --- |
| Nuclease-free Water | 2.5 μl |  | 10 |
| 10X Reaction Buffer | 1.5 μl | 0.75X final | 6 |
| NTP | 1.5 μl each | 7.5 mM each final (4µL total) | 6 |
| Template DNA | 13 μl | ~300-600ng template | 52 |
| T7 RNA Polymerase Mix | 1.5 μl |  | 6 |
| Total reaction volume | 20 μl |  | 80 |

**Table 3:** MasterMix for IVT to make Synthetic Spike S2

**Post-IVT Purification**

1. After IVT, weDNAse treated the reactions by adding 1uL of DNase (NEB, M0303S) to each reaction
2. Incubate reactions at 37°C for 10 minutes.
3. Add 1 µl of 0.5 M EDTA (to a final concentration of 5 mM).
4. Heat inactivate at 75°C for 10 minutes.
5. RNA is purified using the [Zymo RNA clean and concentrator column](https://www.zymoresearch.com/collections/rna-clean-concentrator-kits-rcc).
6. RNA is quantified with an [Agilent TapeStation](https://www.agilent.com/en/product/automated-electrophoresis/tapestation-systems/tapestation-rna-screentape-reagents/rna-screentape-analysis-228268) or Qubit using the RNA BR Kit.
7. **SYNTHETIC S2 SPIKE DILUTION PROTOCOL**

The Synthetic S2 spike is an internal well control for the S2 primer pair. This spike is placed directly in the mastermix and diluted to the same copy number in every single well. Therefore, even for samples in which there is no SARS-CoV-2 virus present, we have an internal control demonstrating that the reaction conditions were sufficient for amplification with the S2 primer pair. The key for the synthetic S2 spike is to include it at a concentration of 100-1000 copies per reaction. Therefore, quantitation of the synthetic S2 spike is key to this experiment-- if the quantitation is off by an order of magnitude, the synthetic spike can overwhelm signal seen in the experiment. We have successfully used 100- and 500-copies per reaction and had successful sequencing runs.

Due to the sensitivity of the spike dilution quantitation, we suggest making aliquots every month and performing RT-qPCR to quantify the copies/reaction. Aliquots are then created from this batch and stored at -80C to minimize freeze thaws.

Protocol for Creating Spike Dilutions:

This should be done fresh every month to prevent degradation of the spike dilutions. Each month, enough aliquots should be created to last the estimated number of runs for the month.

1. Clean Biosafety Hood with 10% bleach and treat bench with UV light for 15 minutes
2. Treat pipettes, 0.1% Tween-20 in 1xTE Buffer, and water with UV light for 15 minutes
3. Dilute and aliquot spike dilution into single use tubes:
   1. Measure the concentration of the S2 stock using HS RNA Qubit.
   2. Using the concentration from the previous step, calculate copies/uL using the equation found using the NEB Bio Calculator (nebiocalculator.neb.com). RNA length is 130 nt.
   3. Perform serial dilutions of 100-fold each using 0.1% Tween-20 in TE until a concentration of ~10,000 copies/uL is reached. Save an aliquot of each dilution for quantification in step 5.
   4. Aliquot the final dilution into individual tubes containing 10uL each. These will be used to spike the master mix for each run of Swabseq.
4. *Perform a Qubit analysis on the first 100-fold dilution to validate.*
5. QC via RT-qPCR and validate copies/uL using “qPCR Validation” in the worksheet linked above. Ct for 100 copies should come up between 33 and 34; for 1000 copies should come up around Ct of 30.
6. **EXTRACTION FREE SAMPLE PROCESSING**
   1. **Saliva Specimen Processing**
      1. Fresh Saliva is collected into a pre-barcoded tube by the user through a plastic funnel. All samples are stored and transported at 4C.
      2. Tubes with samples are placed in 96 well racks in 95C oven in a water bath for 30 minutes.
      3. Dilute 10x TBE solution with sterile and nuclease free water to 2x TBE. Add Tween-20 to a final concentration of 1% Tween. This solution is 2xTBE with 1% Tween used in step 5 and can be stored at room temperature for up to 1 year.
      4. Tubes are spun in 4C centrifuge for 20 seconds at 200xg to collect saliva at bottom of tube.
      5. Rack is placed in the automated decapper (Brooks Intellecap or ThermoFisher Capit-All) and the 96 wells are uncapped.
      6. Using the Benchsmart 96, we pipet 3.5 uL of 2xTBE with 1% Tween-20, pipet an airgap of 2 uL, and then pipet 3.5 uL of saliva directly from tubes using the lowest velocity setting for uptake followed by a second airgap of 2uL. The total volume is dispensed into a designated quadrant of a 384-well plate pre-loaded with primer and mastermix.
      7. After each quadrant is dispensed into the 384-well plate, the 384 well plate is placed into the thermocycler and run with the RT-PCR reaction described below.
   2. **Extraction Free Nasal Swab processing in Normal Saline**
      1. Extraction Free Nasal Swab Specimens are NP, Mid-Nasal and Anterior Nares samples collected directly into 500uL of normal saline.
      2. Tubes are stored and transported at 4C.
      3. Tubes are placed into a 96 well rack and placed in 95C oven in water bath for 30 minutes.
      4. Samples are spun in 4C centrifuge for 20 seconds at 200xg to collect nasal swab sample at bottom of tube.
      5. Rack is placed in the automated decapper (Brooks Intellecap or ThermoFisher Capit-All) and the 96 wells are uncapped.
      6. Using the Benchsmart 96, we pipet 5.6 uL of Nuclease Free water, pipet an airgap of 2 uL, and then pipet 1.4 uL of Extraction Free Nasal Swab followed by a second airgap of 2uL. The total volume is dispensed into a designated quadrant of a 384-well plate pre-loaded with primer and mastermix.
      7. After each quadrant is dispensed into the 384-well plate, the 384 well plate is placed into the thermocycler and run with the RT-PCR reaction described below.
   3. **Extraction Free Nasal Swab processing in TE buffer**
      1. Extraction Free Nasal Swab Specimens are NP, Mid-Nasal and Anterior Nares samples collected directly into 500uL of TE buffer.
      2. Tubes are stored and transported at 4C.
      3. Tubes are placed into a 96 well rack and placed in 95C oven in water bath for 30 minutes.
      4. Samples are spun in 4C centrifuge for 20 seconds at 200xg to collect nasal swab sample at bottom of tube.
      5. Rack is placed in the automated decapper (Brooks Intellecap or ThermoFisher Capit-All) and the 96 wells are uncapped.
      6. Using the Benchsmart 96, we pipet 3.5 uL of Nuclease Free water, pipet an airgap of 2 uL, and then pipet 3.5 uL of Extraction Free Nasal Swab followed by a second airgap of 2uL. The total volume is dispensed into a designated quadrant of a 384-well plate pre-loaded with primer and mastermix.
      7. After each quadrant is dispensed into the 384-well plate, the 384 well plate is placed into the thermocycler and run with the RT-PCR reaction described below.
7. **RT-PCR**

**Make Master Mix** (This calculation is for a single 384 well plates)

Reagents:

TaqPath™ 1-Step RT-qPCR Master Mix (Fisher Scientific, A15300)

NUCLEASE-FREE WATER (Thermofisher, AM9937)

S2 Spike Dilution (see section G)

| **Mastermix Calculation** | | | |
| --- | --- | --- | --- |
|  |  |  |  |
| **per 384 well plate** |  | | |
|  | **RT-PCR mix:** | **uL or copies per reaction** | **Total** |
|  | 4x Mastermix | 5 uL | 1920 |
|  | Water | 6 uL | 2304 |
| Dilution 4 | S2 RNA spike quant  500 copies *384 | 192,000 copies | Calculate from dilution |
|  | | | |
| Sample | 7 | |  |
| indexed primers | 2 | |  |
| Total Volume | 20 | | |
| Total Mastermix per well | 11 | | |

Notes:

- Master mix is made in a clean hood that has been treated for 15 minutes UV light and cleaned with 10% bleach solution.
- All pipettes and water used will be treated with UV light

**Tracking sample plate (refer to sample set up sheet)**

1. For each 96-well plate of 96-rack of matrix tubes, positions A1 and H12 should be left for positive and negative controls.
2. Each plate will be designated a quadrant: 1, 2, 3 or 4 of the 384-well plate. Record the Barcode and quadrant for each 96-well plate or tube-rack of samples.

**Putting Together PCR Plate**

1. Clean benchtop surface with 10% Bleach
2. RNAse inhibitor treatment of benchtop surface
3. Take out pre-stamped primer plates (see section D) from -20 freezer and thaw
4. Spin plates down at 2000xg for 1 minute
5. Visually inspect to ensure there is primer in each well
6. Pipet 11 uL of master-mix into each well
7. Add 7ul of sample from sample plate or tubes into designated quadrant of 384 well plate.

**Reverse Transcription and PCR**

1. Program the following into the 384 Well Veriti Thermocycler

| 1 | 55C | 10 min |
| --- | --- | --- |
| 2 | 95C | 1 min |
| 3 | 95C | 10 sec |
| 4 | 60C | 30 sec |
|  | Go to step 3, 40 cycles |  |
|  | Hold at 12C |  |

1. Load plate into 384 well cycler
2. Press Start

#

# **I. POST-PCR PROCESSING**

# **A. PCR products Combined and purified.**

1. Use Integra Viaflow, pipet 6 ul from each well of a 384 well plate into a sterile reservoir.
2. Repeat step one for each 384 well plate that will be combined into a sequencing reaction run. All samples will be combined into the same reservoir.
3. Slightly tilt the reservoir back and forth to mix.
4. Transfer the entire volume to a 15 mL conical tube and vortex thoroughly.
5. Transfer 100 ul to an eppendorf tube.

# **B. Bead Cleanup**

1. Add 50 ul of AmpureXP beads (0.5:1 ratio of beads: sample volume) to 100 ul volume of pooled PCR reaction. Vortex and let sit for 5 minutes at room temperature.
2. Use magnet to collect beads for 1 minute.
3. Transfer supernatant (~150 ul) to a new eppendorf tube.
4. Add 130 ul of AmpureXP beads to the 150 ul of supernatant. Vortex and let sit for 5 minutes at room temperature.
5. Use magnet to collect beads for 1 minute.
6. Remove liquid and wash beads twice with 500 ul of freshly made 70% EtOH.
7. Elute in 40 ul of qiagen EB buffer.
8. Use a magnet to collect beads for 1 minute. Transfer 33 ul to new eppendorf tube.

# **C. Library Quantification and Quality Control**

1. Make a 1:10 dilution of your eluted library.
2. Use High Sensitivity DNA Qubit to measure the concentration of this 1:10 dilution.
3. Use the following link to determine the concentration of your 1:10 dilution in nM -<https://support.illumina.com/bulletins/2016/11/converting-ngl-to-nm-when-calculating-dsdna-library-concentration-.html>. Use 170 bp for the size.
4. Based on your calculation, make a 7 nM dilution from your 1:10 dilution.
5. Measure this 7 nM dilution and a stock of Illumina phiX using High Sensitivity DNA Qubit.
6. Use the link from step 3 to calculate the concentration in nM of your 7 nM dilution and the Illumina phiX stock.
7. Make a dilution of your phiX stock to equal the nM concentration of your 7 nM dilution. For example, if your “7 nM” dilution was actually 6.39 nM, you would dilute your phiX down to 6.39 nM.
8. Combine 14 ul of your “7 nM” dilution with 6 ul of your dilution of phiX. This results in a “7 nM” library (6.39 nM in our example) that is 30% phiX and 70% our library of interest.
9. Make a 1:2 dilution of your 7 nM dilution and run it on a Agilent Technologies d1000 High Sensitivity Screentape.

**MiSeq run**

**J. PRIMER MIXES FOR SEQUENCING**

1. **MiSeq Primer Mixes**
   1. Add 80 ul of water, 10 ul of S2 read 1 primer (100 uM stock), and 10 ul of RPP3 read 1 primer (100 uM stock) to an eppendorf tube labeled "Read 1 primer mix". Final concentration will be 20 uM of primers (10 uM of each read 1 primer).
   2. Add 80 ul of water, 10 ul of S2 i7 primer (100 uM stock), and 10 ul of RPP3 i7 primer (100 uM stock) to an eppendorf tube labeled "i7 primer mix". Final concentration will be 20 uM of primers (10 uM of each i7 primer).
2. **MiSeq Sequencing**
   1. Load 30uL of the read 1 primer mix into reservoir 12.
   2. Load 30uL of the i7 primer mix into reservoir 13.

Using the determined proportion of library that contains your amplicons of interest, calculate what loading concentration is required to load 28 pM of your amplicons of interest. If 60% of your library is made up of your amplicon of interest, you would load 46.6 pM (28/0.60 = 46.6) of your library on the MiSeq.

1. **NextSeq Primer Mixes**
   1. Add 80 ul of water, 10 ul of S2 read 1 primer (100 uM stock), and 10 ul of RPP3 read 1 primer (100 uM stock) to an eppendorf tube labeled "Read 1 primer mix". Final concentration will be 20 uM of primers (10 uM of each read 1 primer).
   2. In addition to the read 1 primer and i7 primer mix above, the NextSeq requires an i5 primer mix. To make this, add 80 ul of water, 10 ul of S2 i5 primer (100 uM stock), and 10 ul of RPP3 i5 primer (100 uM) stock to an eppendorf tube labeled “i5 primer mix”. Final concentration will be 20 uM of primers (10 uM of each i5 primer).
2. **Next Seq Sequencing**
   1. Load 52uL of the read 1 primer mix into reservoir 20.
   2. Load 85uL of the i7 primer mix into reservoir 22.
   3. Load 85uL of the i5 primer mix into reservoir 22.
   4. Load 1.5 pM of your amplicon library on the NextSeq.

**K. ANALYSIS**

Illumina BCL files are downloaded and converted into FASTQ sequencing files using Illumina’s bcl2fastq software. Each amplicon sequence consists of a set of three individual reads: one 26 base pair read (read1) that identifies the amplicon (S2, S2-spike, or RPP30) and two 10 base pair index reads (index1 and index2) that together uniquely identify the sample. Sequences are assigned to samples using the two index reads and the sum of the reads for each amplicon in each sample is obtained. Decisions about whether the sample passed QC and whether SAR-CoV-2 was detected in a sample are based on the count of sequences observed for each amplicon within each sample and explained in detail below.

**MiSeq Control Software:**

The MiSeq Control Software (Illumina Inc., ‘For Research Use Only’) controls the flow cell

stage, temperature and fluidics system. It also captures images of clusters, generating image analysis, base calling, and base call quality data.

**Real Time Analysis Software:**

Primary analysis is performed by the Real Time Analysis (RTA) software (Illumina Inc., ‘For

Research Use Only’) and consists of base calling of each cluster at each cycle. In addition to basecalling, RTA assigns an analytical quality score (Q-score) to each base call. Calculations of

Q-scores are based on the ratio of the signal intensity of the highest base in a given cluster during a given cycle to the signal intensity of the three other bases. The quality score Q is calculated as -10 log10 P, where P is the probability that base call is incorrect. A minimum of 80% of basecalls must meet the Q30 threshold to proceed to data analysis. If these criteria are not met this could be due to a lack of sequence diversity (insufficient PhiX concentration), a technical problem in library construction, a faulty flow cell or sequencing instrument failure. If fewer than 80% of basecalls meet the Q30 threshold the entire run is discarded.

**bcl2fastq Conversion Software:**

The bcl2fastq conversion software (Illumina Inc., ‘For Research Use Only’) is used to process

BCL (base call log) files generated by the MiSeq instrument and convert time into FASTQ files. FASTQ is a standard text-based file format that will store the nucleotide sequences and base quality scores for each read sequenced from a sample. Three FASTQ files are generated, one corresponding to 26 base pairs of sequence within each amplicon (read1) and two 10 base pair index sequences (index1 and index2) that together uniquely identify each sample.

**UCLA Sample Demultiplexing and Amplicon Counting Software:**

Read1 is matched to one of the three expected amplicons allowing for the possibility of a single nucleotide error in the amplicon sequence. The set of three reads is discarded if read1 has a hamming distance greater than 1 from the expected amplicons. Samples are demultiplexed using the two index reads. Demultiplexing means assigning the sequences to the sample from which they originated. Observed index reads are matched to the expected index sequences allowing for the possibility a single nucleotide error in one or both of the index sequences. The set of three reads are discarded if both index1 and index2 have hamming distances greater than 1 from the expected index sequences. The sum of reads for each amplicon and each sample is calculated.

Scripts detailing our Amplicon Counting Software can be found at <https://github.com/joshsbloom/swabseq>

**L. RESULT INTERPRETATION FOR NP PURIFIED SWABS**

We require that 10 reads are detected for RPP30 for each sample. This serves as a control that sample collection took place properly and contains a human specimen. If fewer than 10 reads are detected for RPP30 the results are considered inconclusive.

We require that the sum of S2 and S2 synthetic spike-in reads exceeds 2,000 reads or the results are considered inconclusive. The S2 synthetic spike-in is added to the master mix, is present in every well and every sample in our assay, and even if no virus is present, if the primers and the assay are working properly the S2 synthetic spike-in will amplify and be sequenced. We have observed that samples with very high viral concentrations will result in high S2 reads and low S2 synthetic spike-in reads, and samples with low viral concentrations will result in low S2 reads and high S2 synthetic spike-in reads. In both cases the sum of S2 and S2 spike should be high in any sample regardless of the presence of Sars-CoV-2. This follows from the fact that the same S2 primers have equal preference for the S2 and S2 synthetic spike and amplify both with equal efficiency.

Assuming a sample as greater than 10 RPP30 reads and that the sum of S2 and S2 synthetic spike-in reads exceeds 2,000, we determine if SARS-CoV-2 is detected in a sample by seeing if the ratio of S2 to S2 spike exceeds 0.003. (We note that we add 1 count to both S2 and S2 spike before calculating this ratio to facilitate plotting the results on a logarithmic scale.) If the ratio is greater than 0.003 we concluded that Sars-CoV-2 is detected for that sample and if it is less than or equal to 0.003 we conclude that Sars-CoV-2 is not detected.

| **Well-controls** | |  | **Results** | | | |
| --- | --- | --- | --- | --- | --- | --- |
| **Total S2 + S2 Spike** | **RPP30 read count** |  | **S2/S2 spike ratio** | **Result** | **Interpretation** | **Action** |
| >2000 reads | >10 |  | > 0.003 | SARS- CoV-2  Detected | Positive for SARS-CoV-2 for the Sample ID. | Report results to physician, patient, and appropriate public health authorities. |
| >2000 reads | >10 |  | < 0.003 | SARS- CoV-2  Not Detected | Negative for SARS-CoV-2 for the Sample ID. | Report results to physician, patient, and appropriate public health authorities. |
| <2000 reads | >10 |  | - | Inconclusive | Invalid for the Sample ID. | Quality control for the Sample ID is FAIL. Repeat sample or Recollect sample |
| >2000 reads | < 10 |  | - | Inconclusive | Invalid for the Sample ID. | Quality control for the Sample ID is FAIL. Repeat sample or Recollect sample |
| <2000 reads | < 10 |  | - | Inconclusive | Invalid for the Sample ID. | Quality control for the Sample ID is FAIL. Repeat sample or Recollect sample. |

**RESULT INTERPRETATION FOR EXTRACTION-FREE SALIVA AND NASAL SWAB**

We require that the sum of S2 and S2 synthetic spike-in reads exceeds 500 reads, or the results are considered inconclusive. With inhibitory lysates we have observed that this lower total is acceptable for maintaining sensitivity and specificity while limiting the number of tests that are considered inconclusive. If the sum of S2 and S2 synthetic spike-in reads exceeds 500, we determine if SARS-CoV-2 is detected in a sample by seeing if the ratio of S2 to S2 spike exceeds 0.003. (We note that we add 1 count to both S2 and S2 spike before calculating this ratio to facilitate plotting the results on a logarithmic scale.) If the ratio is greater than 0.003 we concluded that Sars-CoV-2 is detected for that sample and if it is less than or equal to 0.003 we conclude that Sars-CoV-2 is not detected as long as 10 reads are detected for RPP30 for that sample. This serves as a control that sample collection took place properly and contains a human specimen. If fewer than 10 reads are detected for RPP30 and the ratio of S2 to S2 spike is less than or equal to 0.003 the results are considered inconclusive. We have modified this criteria such that only samples without Sars-CoV-2 signal are considered inconclusive if RPP30 reads are fewer than 10. This ensures that we do not miss Sars-Cov-2 positive samples that may have fewer RPP30 reads.

| **Well-controls** | |  | **Results** | | | |
| --- | --- | --- | --- | --- | --- | --- |
| **Total S2 + S2 Spike** | **RPP30 read count** |  | **S2/S2 spike ratio** | **Result** | **Interpretation** | **Action** |
| >500 reads | >0 |  | > 0.003 | SARS- CoV-2  Detected | Presumptive positive for SARS-CoV-2 for the Sample ID. | Report results to physician, patient, and appropriate public health authorities. |
| >500 reads | >10 |  | < 0.003 | SARS- CoV-2  Not Detected | Presumptive Negative for SARS-CoV-2 for the Sample ID. | Report results to physician, patient, and appropriate public health authorities. |
| <500 reads | >0 |  | - | Inconclusive | Invalid for the Sample ID. | Quality control for the Sample ID is FAIL. Repeat sample or Recollect sample |
| >500 reads | < 10 |  | <.003 | Inconclusive | Invalid for the Sample ID. | Quality control for the Sample ID is FAIL. Repeat sample or Recollect sample |

**Appendix A.**

Measuring the Efficiency of amplification of S2 spike amplicon and C19 genomic RNA.


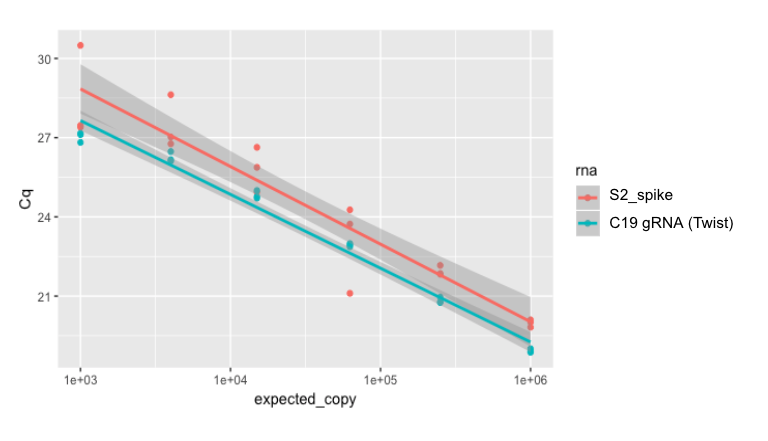


RT-qPCR demonstrates that the amplification efficiency of S2-spike in amplicon is similar to the efficiency of gRNA control for SARS-CoV2 (Twist Biosciences, Control #1)

**Appendix B.**

Quantification of the S2 Spike-In dilutions.


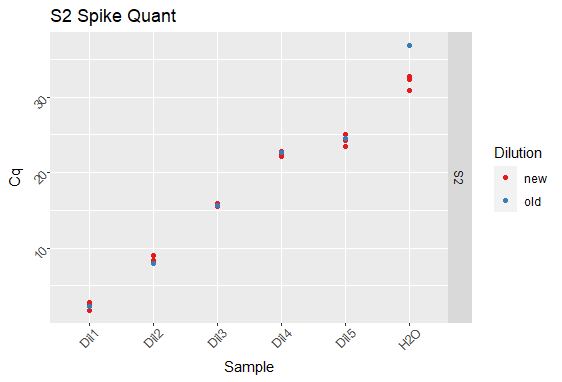


**Appendix C.**

Agilent High Sensitivity D1000 ScreenTape Protocol


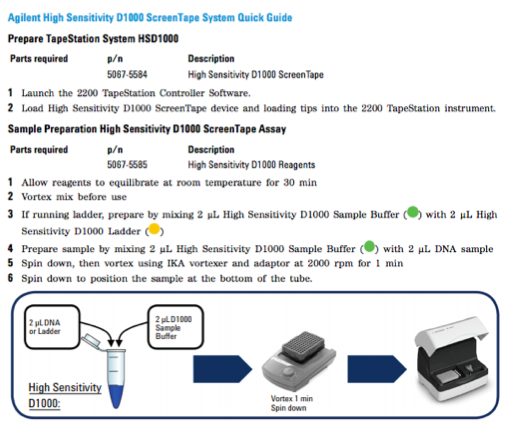


**Appendix E:**

TapeStation Amplicon Quantification Method


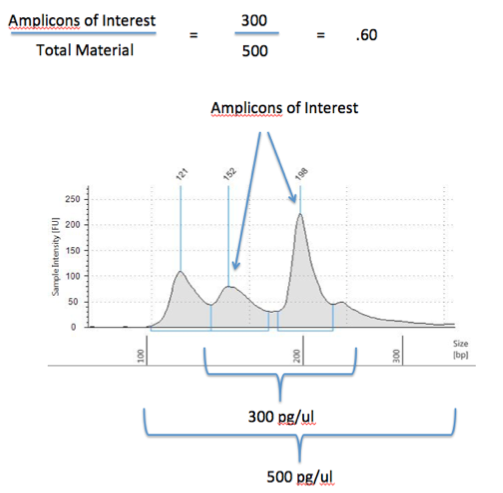


The library trace from the tapestation should look similar to the one above. We are interested in the relative concentration of the peaks at ~150 bp and ~200 bp. Using the “edit region” feature on the tapestation analysis software, you can determine the concentration of specific regions. Use this feature to compare the concentrations of the regions shown above and find what proportion of your library contains your amplicons of interest.

**Appendix F**: Taqman qPCR Protocol

This is a general protocol for running a qPCR using the CDC’s taqman probes.

**Materials:**

- TaqPath Master Mix
- Indexed Primers for S2 (same working concentration as
- UV treated molecular-grade water
- UV treated pipettes and tubes

**Procedure:**

1. Prepare master mix by combining the following for n = number of samples + 3 for each desired probe
   1. 5uL 4x TaqPath master mix
   2. 1.4uL taqman primer
   3. 8.6uL H2O
2. Plate master mix into desired wells
3. Add 5uL of sample to each well
4. On qPCR machine, run plate using the following protocol

| **Stage** | **No. of Cycles** | **Temp (C)** | **Time** |
| --- | --- | --- | --- |
| 1 | 1 | 25 | 2 minutes |
| 2 | 1 | 50 | 15 minutes |
| 3 | 1 | 95 | 2 minutes |
| 4 | 45 | 95  60 | 3 seconds  30 seconds |
| 5 | - | 4 | Infinite hold |

- 1. If desired, perform a melt curve

**Appendix H. Quality control (to avoid and detect any contamination in the assay)**

1. Every qPCR run of 96 samples has NTC (no template control) in well H12.
2. When preparing mastermix, work space and all pipettes are DNA-zapped and UV treated (3 min).
3. When preparing mastermix, empty PCR plates, empty qPCR plates, empty tubes, tips, and water for mastermix are UV-treated.
4. Mastermix preparation occurs in a PCR-free clean hood (no amplified PCR product in this room, no Viral RNA template in this room, and no positive control sample in this room.)
5. Any opened positive control sample is prepared and physically stored in a separate room from where taqman mastermix plates are prepared.
6. No Template Controls (NTC) are prepared in a separate room from where the positive control is stored/prepared. NTC water and tubes are UV-treated before and after preparation.
7. qPCR preparation steps are performed on an automated liquid handling platform. All automation for qPCR preparation is done using disposable, filtered tips.
8. Before manually adding positive and negative controls to the RNA sample plates, bench space and pipettes are DNA-zapped.
9. Plate-seals on extracted RNA sample plates are gently pierced with tips on the automated liquid handler when aspirating RNA and dispensing into the qPCR plates. This eliminates the possibility of spray between sample wells that can occur during manual removal of seals.
10. qPCR occurs in a separate room from mastermix plate creation or RNA sample addition.
11. Each Sequencing run is reviewed by a clinical supervisor before being reported out.
