# Supplementary material for: Swab-Seq: A high-throughput platform for massively scaled up SARS-CoV-2 testing
Source: medRxiv. 2021 Mar 9:2020.08.04.20167874. Preprint. [Version 4] doi: 10.1101/2020.08.04.20167874 (PMC7480060; doi:10.1101/2020.08.04.20167874)
Supplement: 1 [file NIHPP2020.08.04.20167874-supplement-1.pdf]

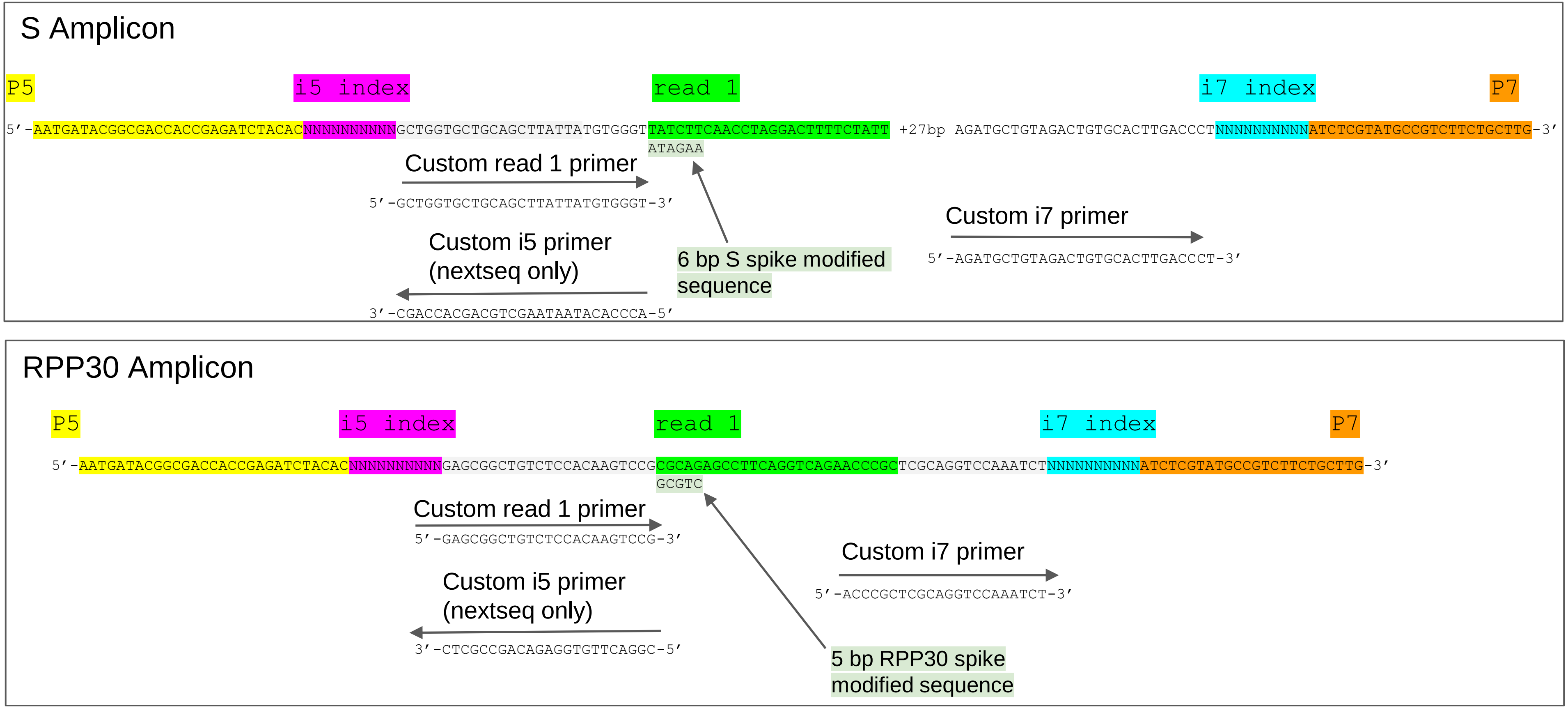

Figure S1. Sequencing library design. The amplicon designs are shown for the S (top) and RPP30 (bottom) amplicons. Amplicons were designed such that the i5 and i7 molecular indexes uniquely identify each sample. SwabSeq was designed to be compatible with all Illumina platforms.

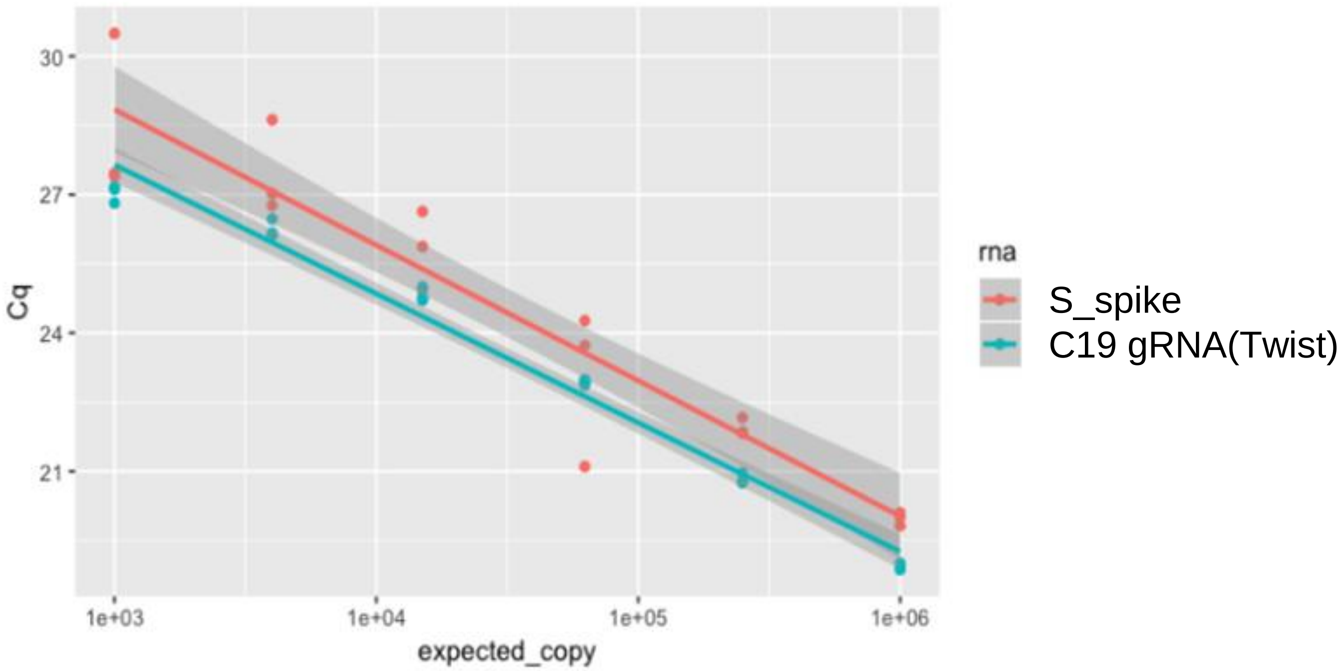

Figure S2. The S primers show equivalent PCR efficiency when amplifying the COVID-19 S gene amplicon and the synthetic *in vitro* S standard. Slope of PCR efficiency of the primers with either the S_spike (labeled in red) or the SARS-CoV-2 viral (labeled in green as C19gRNA) input are as follows: S_spike slope = -6.68e-6 and C19gRNA (Twist Control) slope = -6.74e-6. The slopes are expected to equivalent (parallel) if the primers do not show preferential amplification of the S spike RNA versus the C19gRNA. This shows that the S spike and C19gRNA have equivalent amplification efficiencies using the S primer pair. The bands represent 95% confidence intervals for predicted values, are non-overlapping due to different intercepts, and are not relevant for this analysis of slopes.

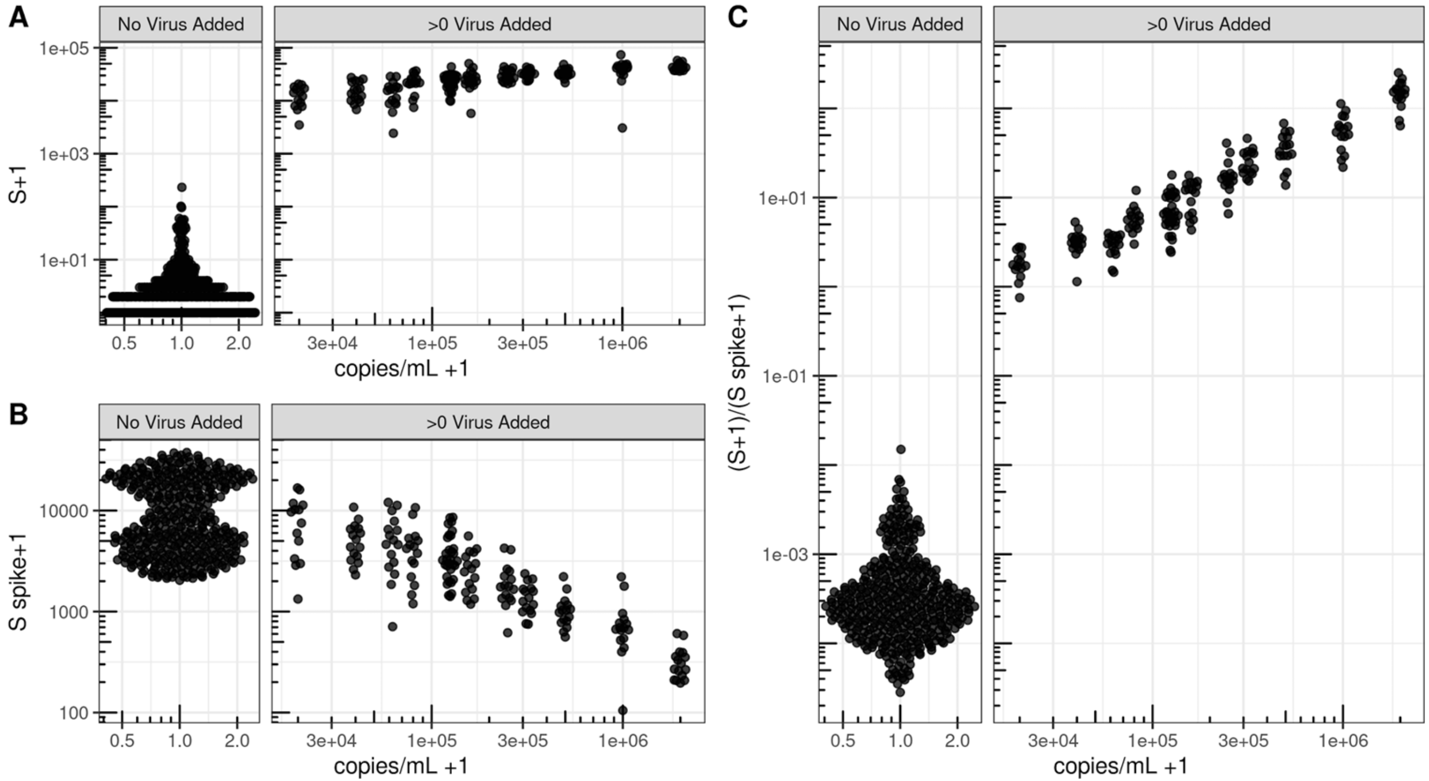

Figure S3. At very high viral concentrations SwabSeq maintains linearity. We include an internal well control, the S Spike, to enable us to call negative samples, even in the presence of heterogeneous sample types and PCR inhibition. (A) As virus concentration increases, we observe increased reads attributed to S and (B) decreased reads attributed to the S Spike. (C) The ratio between the S and S Spike provides an additional level of ratiometric normalization and exhibits linearity up to at least 2 million copies/mL of lysate. Note that ticks on both axes are spaced on a log10 scale.

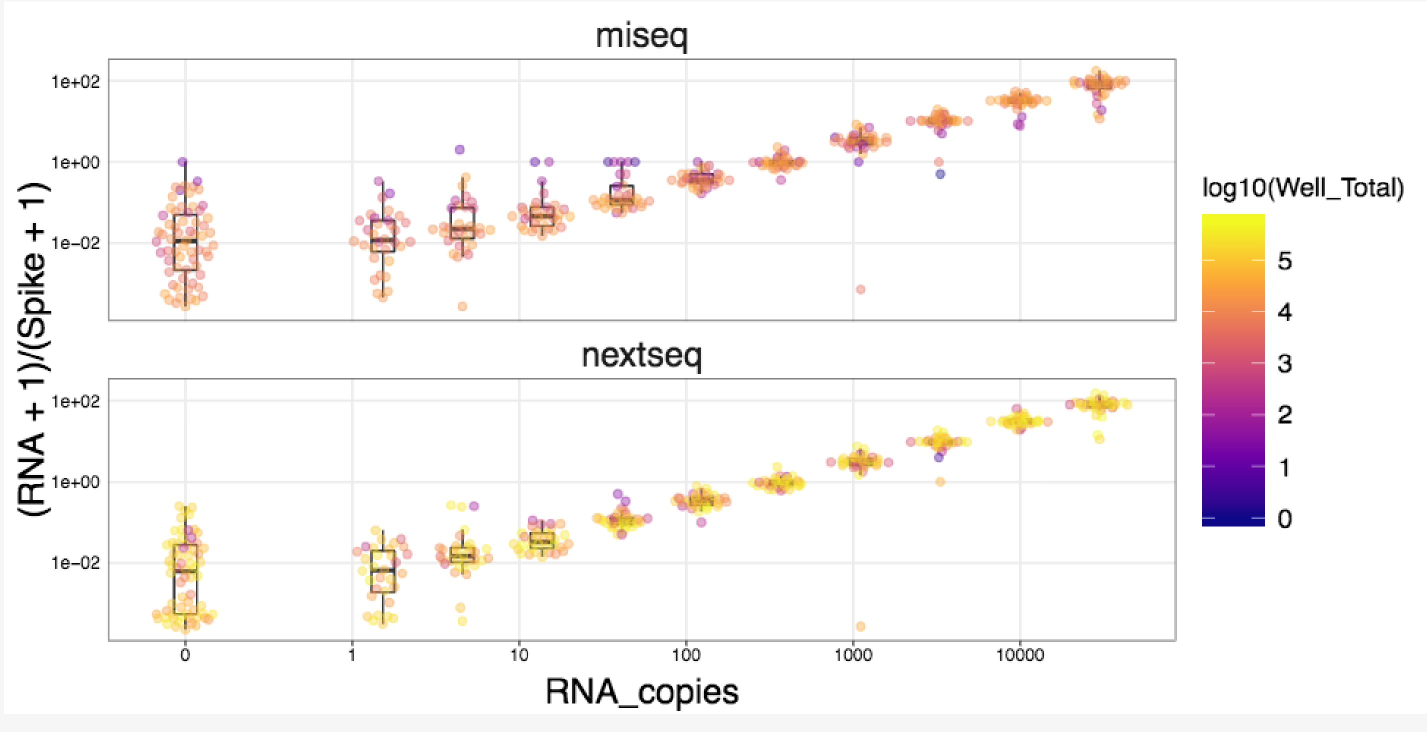

Figure S4. Sequencing is performed on MiSeq or NextSeq Machine with similar sensitivity. Multiplexed libraries run on both MiSeq and NextSeq showed linearity across a wide range of SARS-CoV2 virus copies in a purified RNA background.

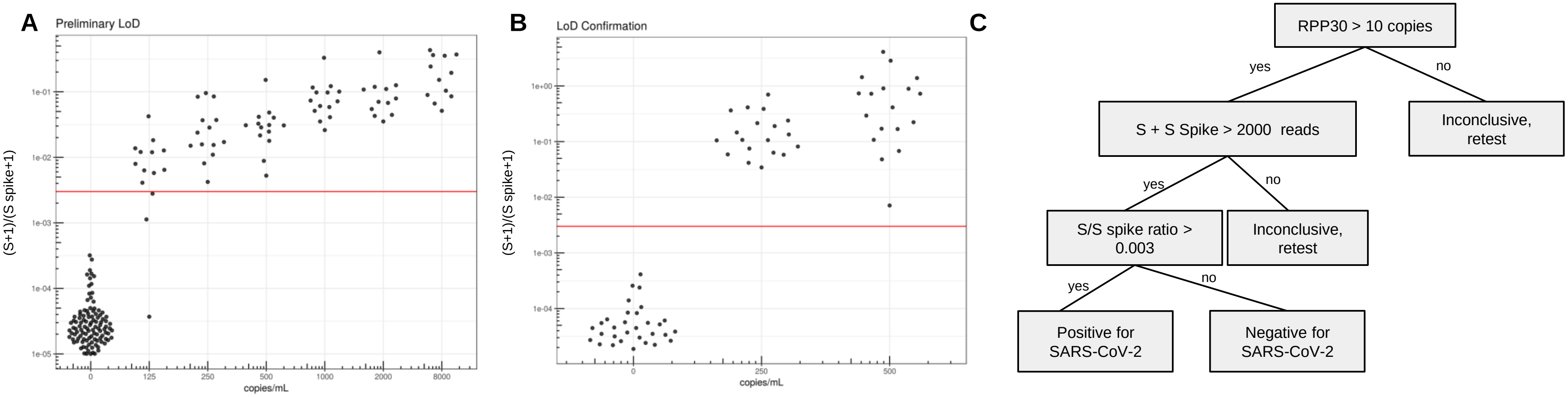

D

		UCLA RT-qPCR		
		**Positive**	**Negative**	
**SwabSeq**	**Positive**	94	0	94
	**Negative**	0	282	282
	**Inconclusive**	0	4	4
	Total	94	286	380
	Positive Percent Agreement			1
	Negative Percent Agreement			98.6%
	Overall Agreement			98.9%

Figure S5. Preliminary and Confirmatory Limit of Detection Data for RNA purified Samples A) Our preliminary LOD data identified a LOD of 250 copies/mL using the NextSeq550 B) Confirmatory studies showed an LOD of 250 copies/mL using the NextSeq550. C) Our result interpretation guidelines for purified RNA. D) Concordance of the 380 clinical samples that were run during our validation process. Concordance is 98.6%, with 100% positive percent agreement (PPA) and 100% negative percent agreement (NPA).

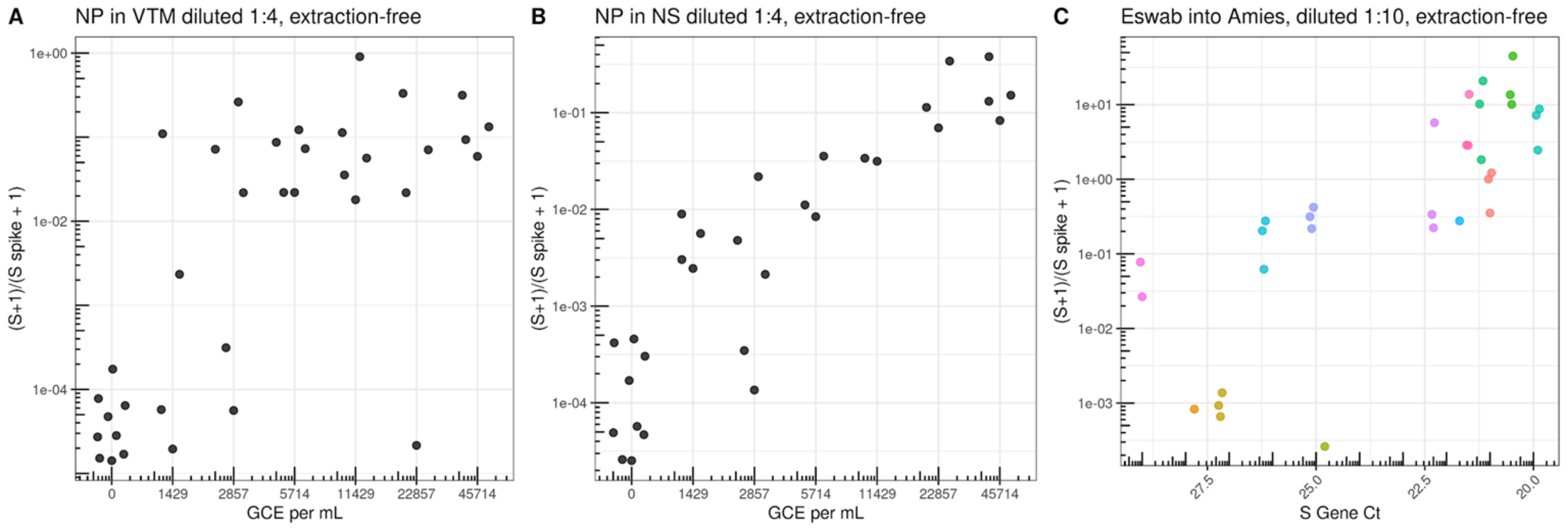

Figure S6. Extraction-Free protocols using traditional collection medias and buffers require dilution to overcome effects of RT and PCR inhibition. A) We tested extraction free protocols for nasopharyngeal swabs that were placed into viral transport media (VTM). We spiked ATCC live inactivated virus at varying concentrations into pooled VTM and then diluted samples 1:4 with water before adding to the RT-PCR reaction. We observed a limit of detection of 5714 copies per mL. B) We also tested nasopharyngeal (NP) swabs that were collected in normal saline (NS), pooled and then spiked with ATCC live inactivated virus at varying concentrations. Contrived samples were diluted 1:4 in water. Here, our early studies show a similar similar limit of detection between 2857 and 5714 copies per mL. C) We tested natural clinical samples that were collected into Amies Buffer (ESwab). Here we compare S gene Ct count (x-axis) from positive samples to the SwabSeq S to S spike ratio (y-axis). Samples were run in triplicate (colors). We observed high concordance for Ct counts of 27 and lower but more variability for Ct counts greater than 27 suggesting that RT and PCR inhibition were affecting our limit of detection. Based on these data we opted only to further explore extraction free protocols into normal saline or tris-EDTA buffer.

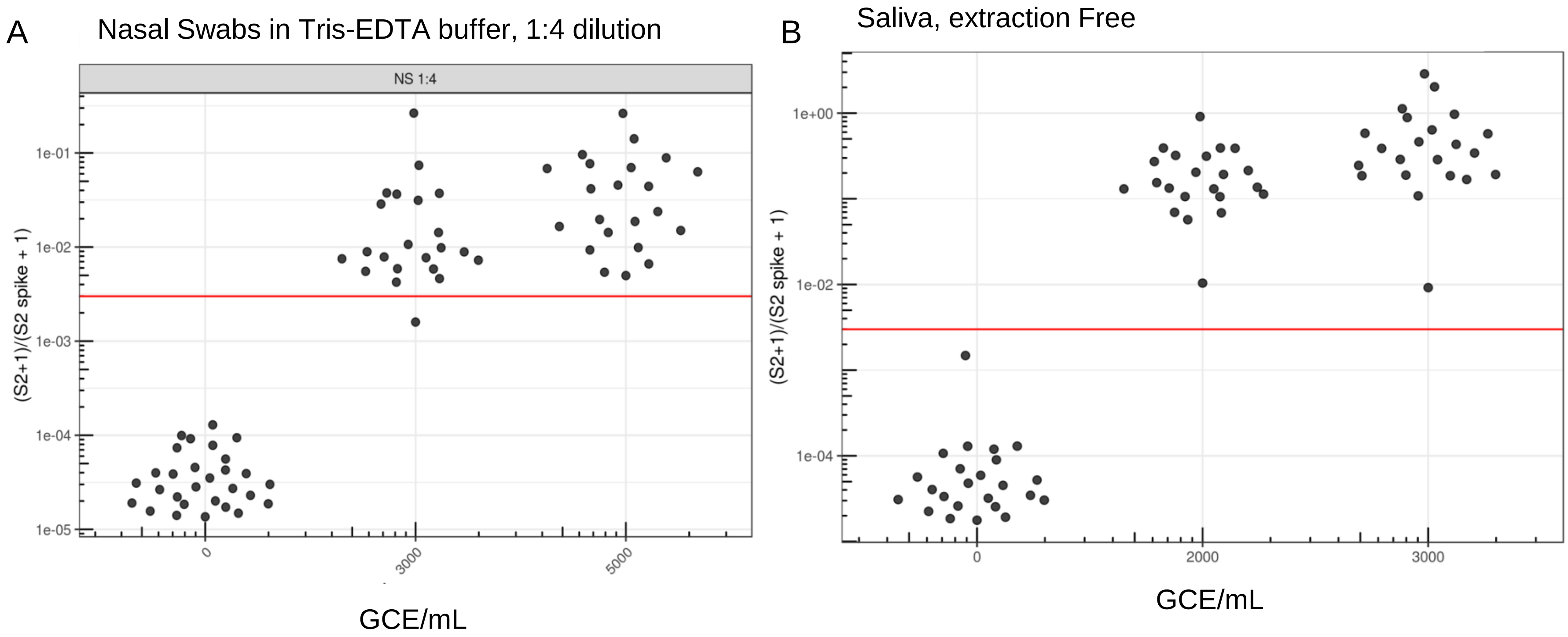

Figure S7. Confirmatory Limit of Detection Data for Extraction Free specimens. A) Our data showed a LOD of 3000 copies/mL for Nasal Swabs samples that were diluted 1:4 in water. These dilutions were performed in replicate at 20 samples per concentration. We tested multiple replicates around the limit of detection. B) Confirmatory studies for extraction-free saliva samples showed an LOD of 2000 copies/mL. Red line indicates the threshold for positivity.

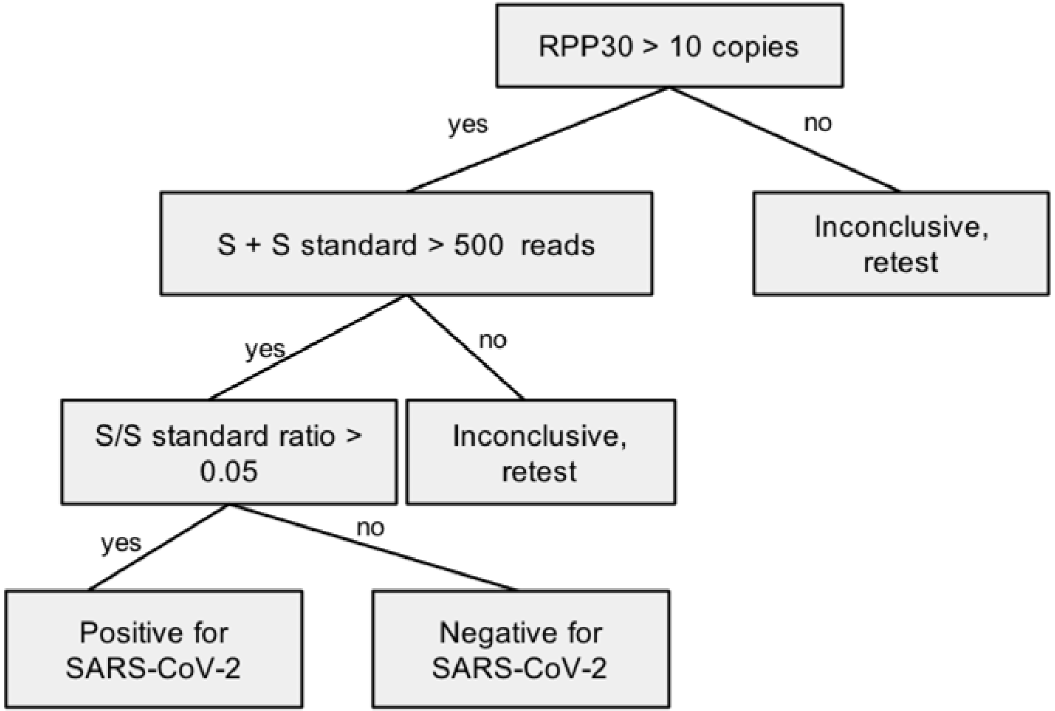

Figure S8. Per sample decision tree for extraction-free samples. Given the slight modifications for our extraction free protocols, we have modified our decision tree to reflect the differences in extraction-free sample types. Our result interpretation guidelines for extraction-free samples relax threshold for S + S spike to 500 reads due to the increased PCR inhibition observed in extraction free sample types. The standard used in our early validations had a slightly lower S/S standard ratio of 0.03 which ultimately in clinical testing had too many false positives. Our current validated test uses the above ratio for extraction free samples.

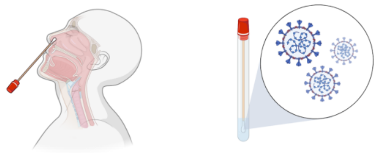

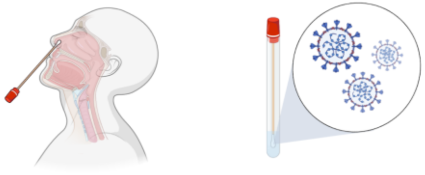

		UCLA RT-qPCR		
		Positive	Negative	
SwabSeq	Positive	80	0	80
	Negative	6	206	212
	Inconclusive	1	5	6
		87	211	298

	Positive Percent Agreement	92.0%
	Negative Percent Agreement	97.6%
	Overall Agreement	96.0%

Figure S9. Comparison of extraction-free NP samples run on SwabSeq to NP Swab samples processed to Clinical pathway using RNA purification and RT-qPCR. Evaluation of extraction free nasal swabs processed into normal saline or Tris-EDTA ph 8.0 that have previously tested positive or negative in the UCLA Clinical Microbiology Laboratory. We have explored the sources of false negatives in our data set. Three of the four false negatives stem from differences in the limit of detection, where we do not always detect samples with Ct > 30.

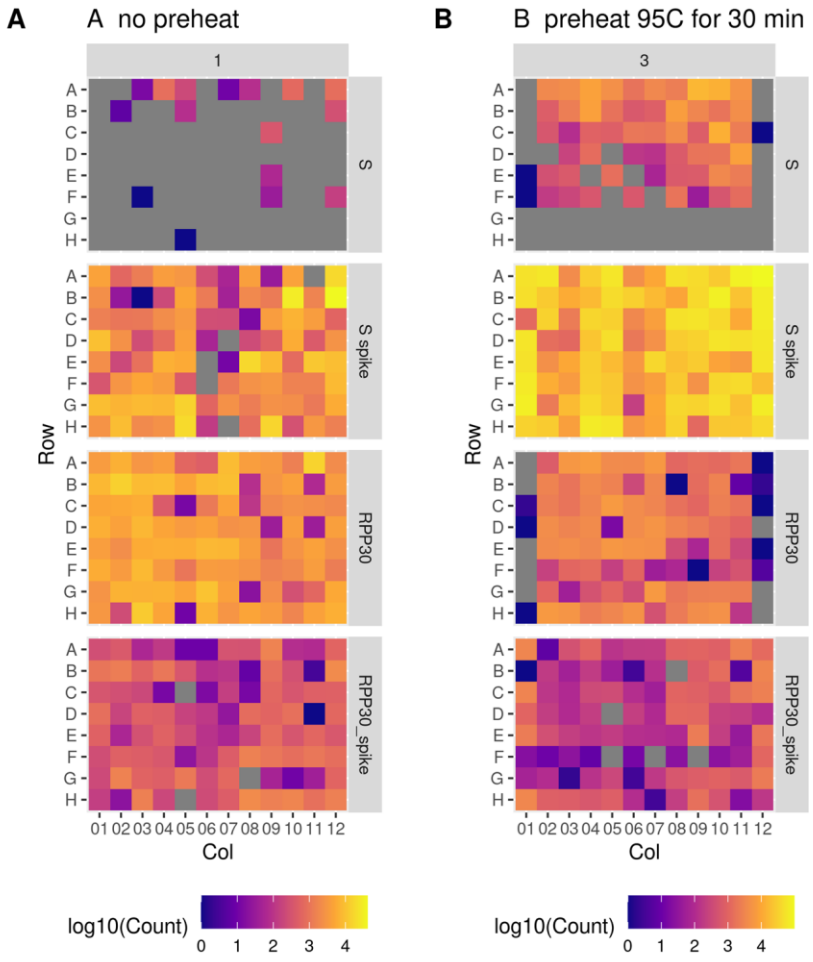

Figure S10. Preheating Saliva to 95C for 30 minutes drastically improves RT-PCR. Detection of viral genome and shows improved robustness in detection of our controls. A) Without preheating, detection of S spike is minimal and there are lower counts for the control amplicons. B) with a 95C preheating step for 30 minutes, we observe robust detection of the S amplicon and synthetic S Spike.

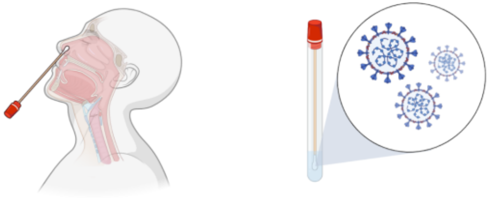

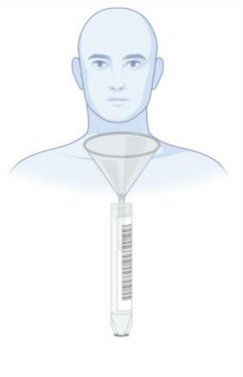

		UCLA RT-qPCR NP Swab		
		Positive	Negative	
SwabSeq Saliva	Positive	74	2	76
	Negative	6	438	444
	Inconclusive	2	15	17
		82	455	537

Positive Percent Agreement	90.2%
Negative Percent Agreement	96.3%
Overall Agreement	95.3%

Figure S11. Comparison of extraction-free saliva samples run on SwabSeq to NP Swab samples processed to Clinical pathway using RNA purification and RT-qPCR. We performed a series of studies to compare the concordance of Saliva and NP swab performed within 2 hours of each other. These collections were obtained in the UCLA ED and UCLA Student Health Center over the course of several months.

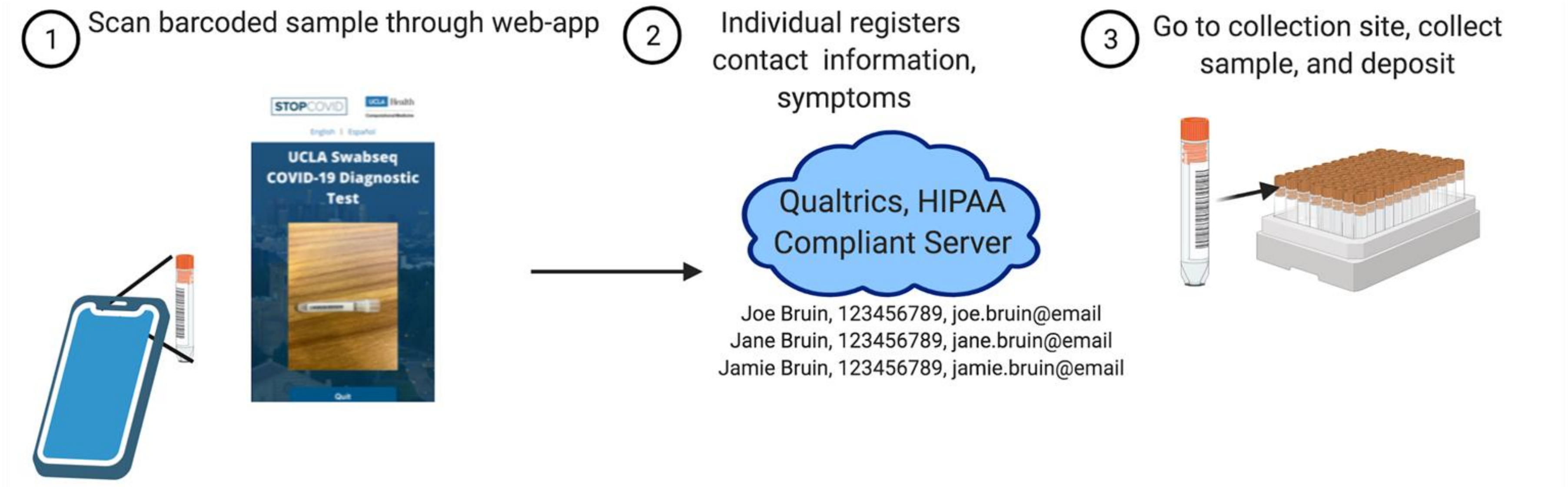

Figure S12. Developing a lightweight sample accessioning to allow for scalable testing into the thousands of samples per day. A major bottleneck is the sample registration to an individual patient. To facilitate the sample accessioning we developed a web-based app for individuals to register their sample tube using a barcode reader and send their identifying information into a secure instance of Qualtrics. In scaled clinical testing in our CLIA laboratory, we used an instance of PreciseQ MDX that allowed organizations to invite cohorts for testing based on their specific needs.

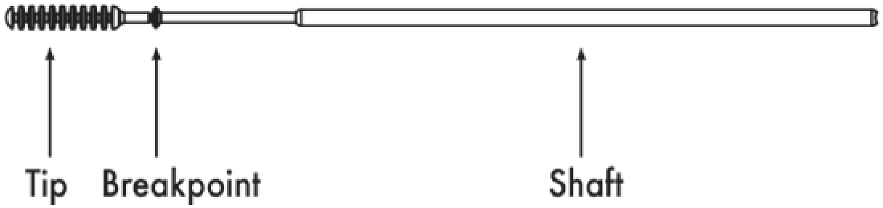

Figure S13. Developing a automation compatible swab. A major bottleneck is the sample processing is the manual process of opening tubes and removing the swab. Not only is this manual but also a source of both cross contamination and biohazardous exposure. To limit this, we designed a 3D printed swab, in conjunction with Applied Medical Company where the breakpoint was engineered to break close to the swab edge such that it would not interfere with our automated pipette machinery.

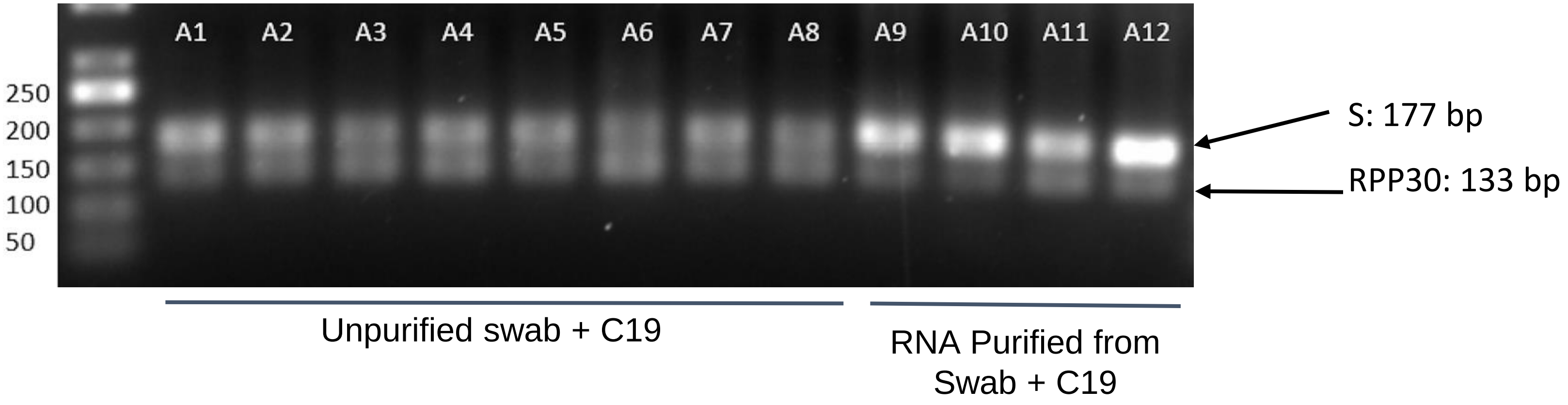

Figure S14. PCR inhibition has significant effect on amplification products. A) 2% Agarose gene was run for a subset of wells from our Rt-PCR reactions. We observe RT-PCR inhibition from swabs in unpurified lysate (A1-A8) as compared to purified RNA (A9-A12). We observe two bands in this subset of wells representing 2 amplicons for the S or S spike (177bp) and RPP30 (133 bp) primer pairs.

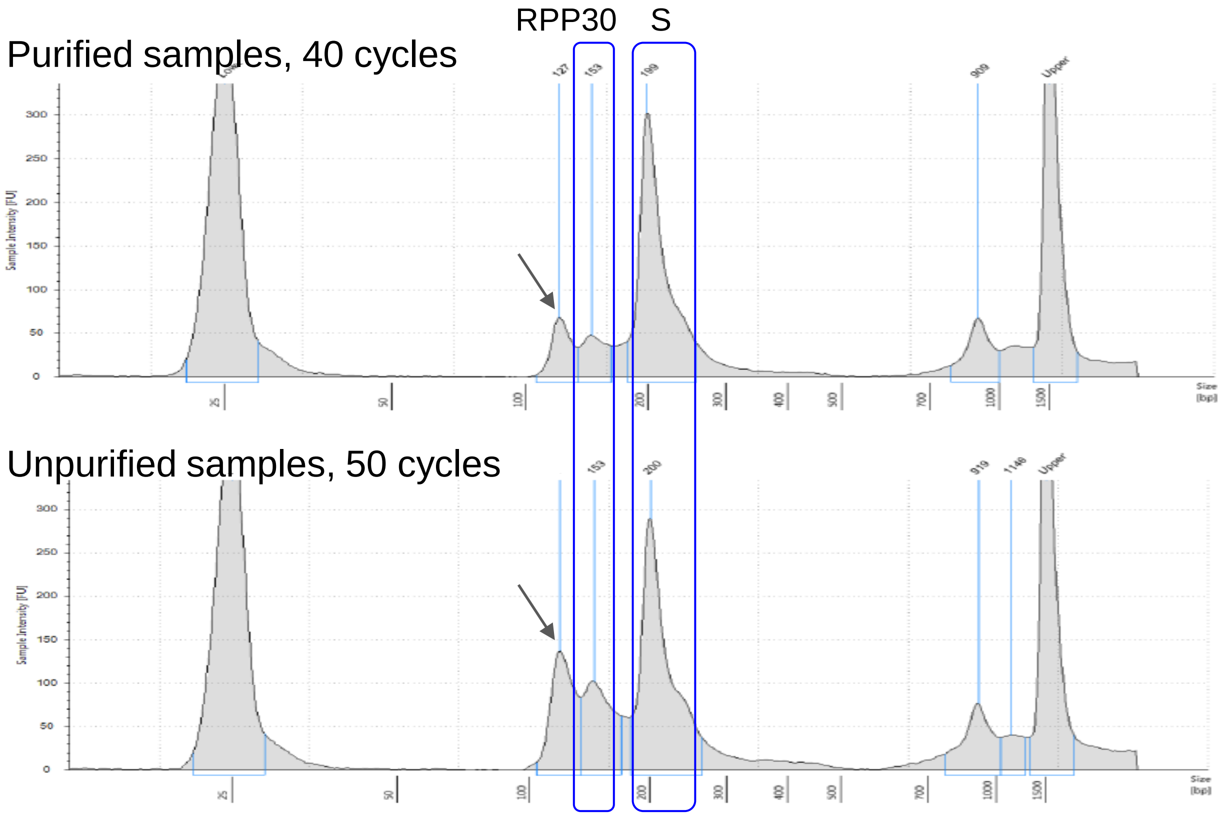

Figure S15. Tapestation Increasing the number of PCR cycles and working with unpurified or inhibitory samples types (eg. Saliva) was seen to increase the size of a nonspecific peak in our library preparation. Representative result from Agilent TapeStation for our purified amplicon libraries. We observe a nonspecific peak slightly above 100bp (arrow) in both library traces, but this peak increases in size with unpurified samples and an increased number of PCR cycles. While we have not confirmed the identity of this peak, we believe this peak may be the result of adapter dimers or unsequenceable PCR artifacts. Importantly, we observe that an increase in the size of this nonspecific peak leads to inaccurate library quantification. Therefore, in order to optimize cluster density on Illumina sequencers, we suggest quantifying the loading concentration of the final library based on the proportion of the desired peaks (RPP30 and S).

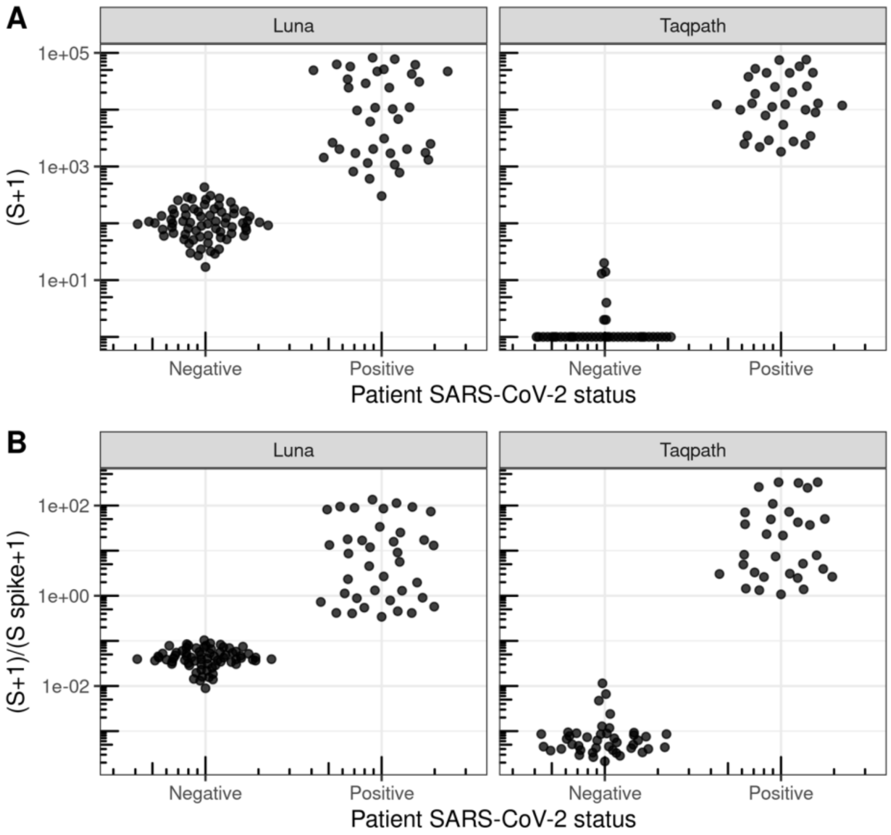

Figure S16. TaqPath decreases the number of S reads in SARS-CoV2-negative samples relative to NEB Luna. We compared Luna One Step RT-PCR Mix (New England Biosciences) to TaqPath™ 1-Step RT-qPCR Master Mix (Thermofisher Scientific). It is likely that the presence of UNG in the TaqPath Mastermix significantly reduced the number of S reads in the SARS-CoV-2-negative samples allowing us to more accurately distinguish SARS-CoV-2-positive and SARS-CoV-2-negative samples.

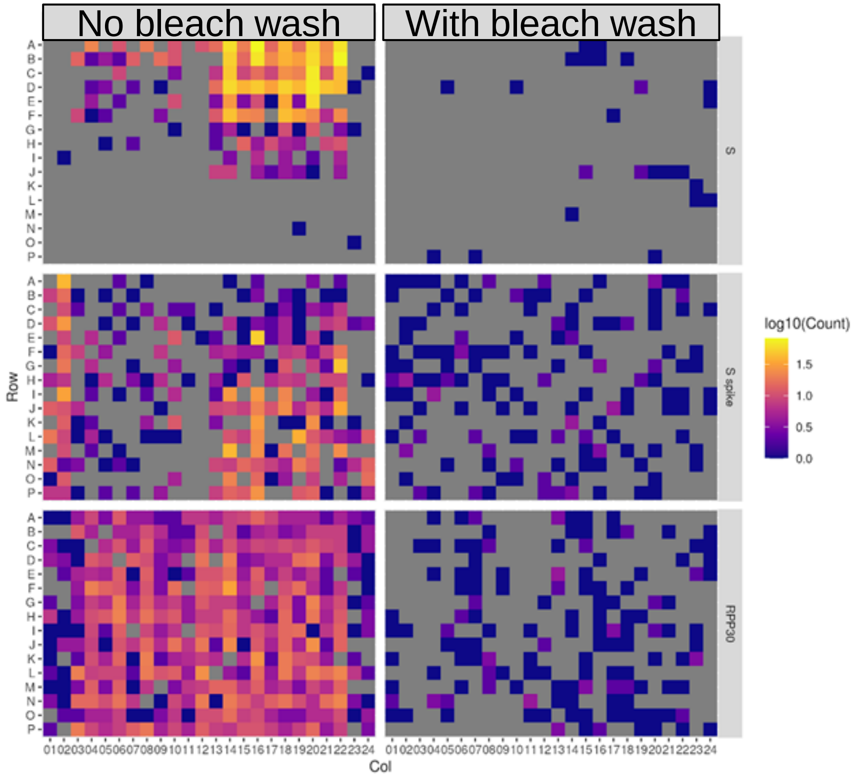

Figure S17. Carryover contamination from template line in a MiSeq contributes to cross contamination. In this experiment we did RT-PCR on four 384-well plates but only pooled three plates. On the left are observed counts of each of the amplicons for each sample for the 384-well plate not included in our run (but for which the indices were used in the previous run). Amplicon reads for indices used in the previous run are present at a low level (0-150 reads). We then performed a bleach wash in addition to regular wash prior to the subsequent run. In this subsequent run, we pooled three different plates and left out the fourth 384 well plate. On the right are observed counts of each of the amplicons for sample indices corresponding to the left-out plate (again, for which the indices were used in the previous run). We observe a remarkable decrease in the amount of carryover contamination, where carryover reads are <10 per sample.

A

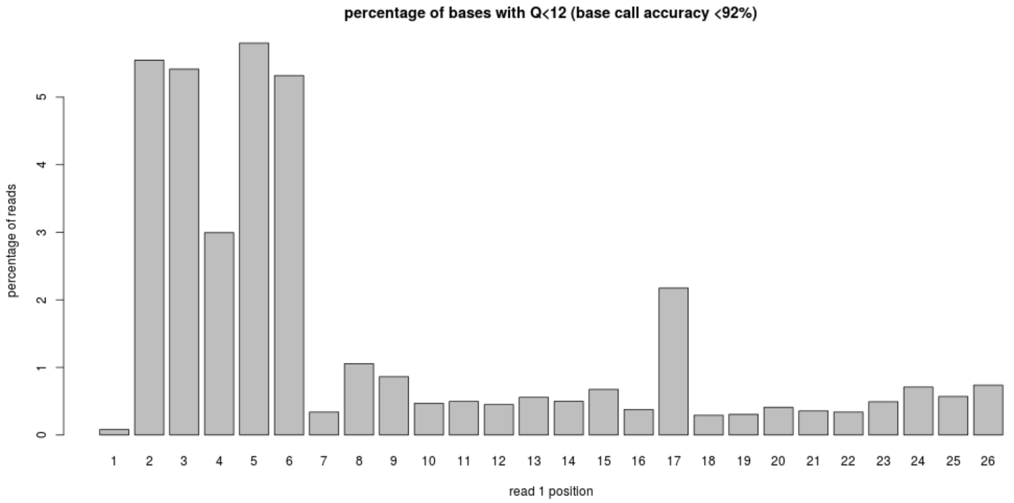

B

Hamming distance from S spike (columns); Hamming distance from S (rows)

	0	1	2	3	4	5	6
**0**	0	0	0	0	0	0	981607
**1**	0	0	0	0	0	49010	235315
**2**	0	0	0	0	22578	19024	111529
**3**	0	0	0	25516	7089	15226	59012
**4**	0	0	74519	8036	7576	10021	36688
**5**	0	522655	21671	9469	3292	5824	13217
**6**	9062990	222210	48991	2270	1337	1669	2140
**7**	0	928836	19700	2439	277	253	348

Figure S18. Sequencing errors in amplicon read and potential amplicon mis-assignment. In experiment v18 we loaded less PhiX than usual (11%) and the overall quality of read1 was lower. Trends noticed here persist in other runs but this run more clearly highlights issues that can occur due to sequencing errors and overly tolerant error-correction. A) The percentage of reads with base quality scores less than 12 for each position in read 1. Note that the first 6 bases of read1 distinguish S from S spike and have the highest percentage of low quality base calls. B) The hamming distance between each read1 sequence and either the expected S sequence (rows) or S spike sequence (columns), In yellow are perfect match and edit distance 1 sequences that can be clearly identified as S or S spike. In red are sequences with errors that may be mis-assigned (S spike assigned as S is most problematic for this assay.)

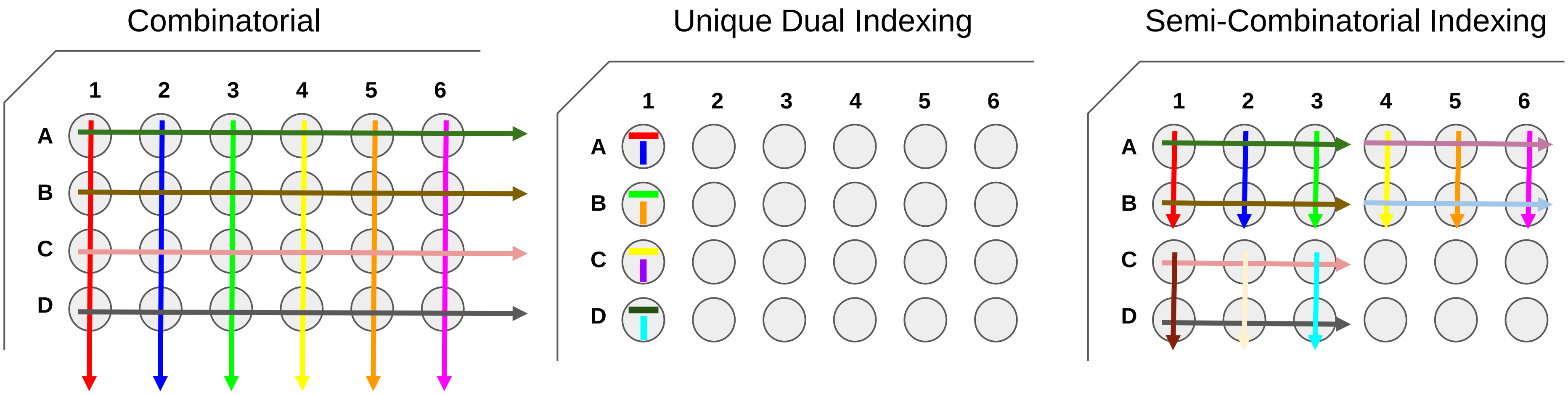

Figure S19. Visualization of different indexing strategies. Here i5 indices are depicted as horizontal lines, i7 indices are depicted as vertical lines, and colors represent unique indices. In combinatorial (or fully-combinatorial) indexing, the i5 and i7 indices are combined to make unique combinations, but each i5 and i7 index may be used multiple times within a plate, and all possible i5 and i7 . For unique dual indexing, each i5 and i7 index are only used 1 time per plate. This requires many more oligos to be synthesized. For Semi-Combinatorial indexing, the combinations used are more limited, such that indices are only repeated for a subset of wells and many possible combinations are not used. In practice (not depicted here), we've used a design where the i7 index is unique but the i5 index can be repeated up to four times across a 384-well plate. For the majority of our SwabSeq development, we used either semi-combinatorial indexing (384x96) that allowed for 1536 combinations or samples to be run or unique dual indexing (384 UDI)

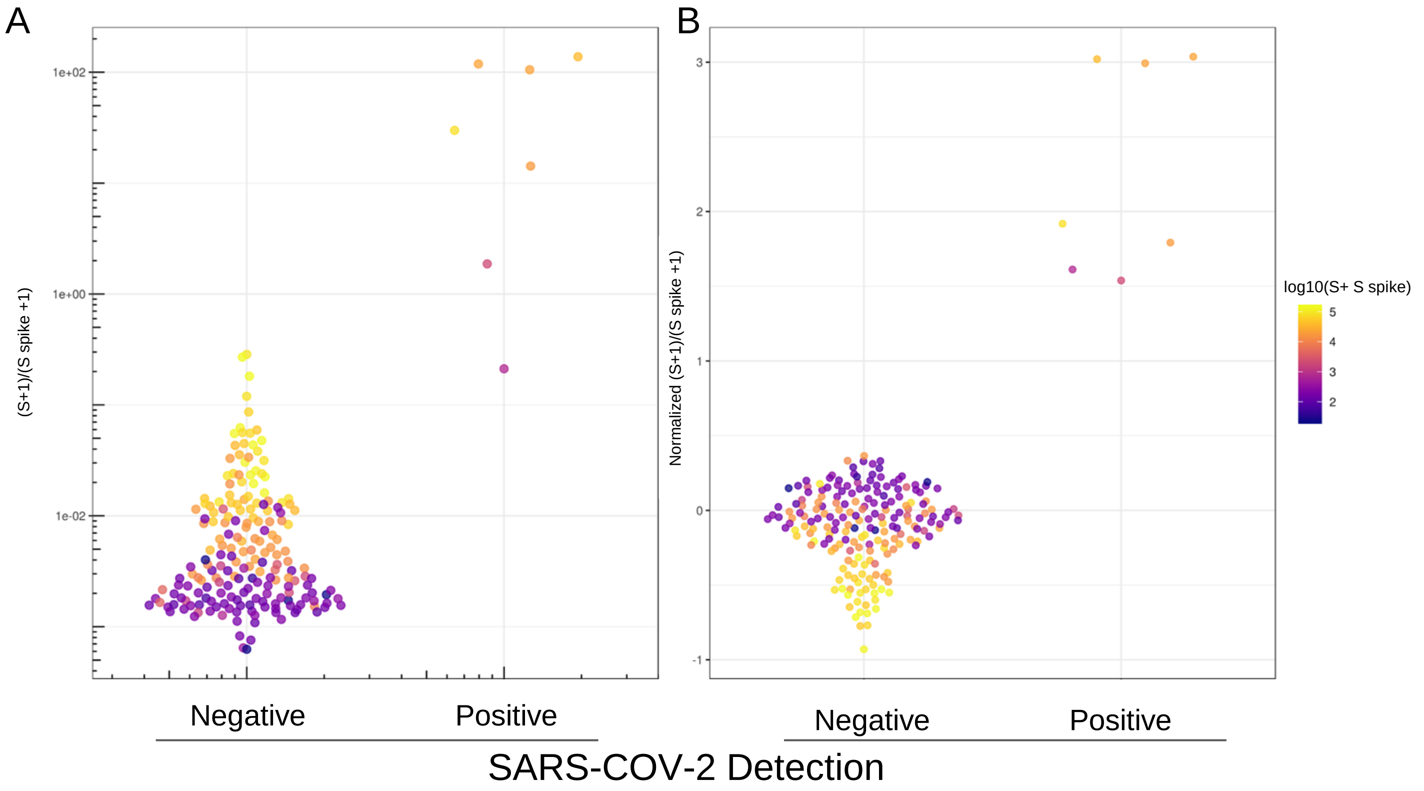

Figure S20. Computational correction for index mis-assignment using a mixed-model. To expand the number of samples we are capable of testing, we can use a combinatorial indexing strategy. In this experiment we used a single index on i5 to uniquely identify a plate and 96 i7 indices to identify wells. (A) The ratio of S to S spike (y-axis) is plotted for clinical samples based on whether Covid was detected by RT-qPCR (x-axis). SARS-CoV-2 positive samples were filtered to have Ct<32. The effects of index mis-assignment across plates can be observed as i7 indices that have high a sum of S and S spike across all samples that share the same i7 barcode across plates (colors). (B) Best linear unbiased predictor residuals are plotted (y-axis) for data in A, after computational correction of the log10(S+1/S spike+1) ratio by treating the identity of the i7 barcode as a random effect.

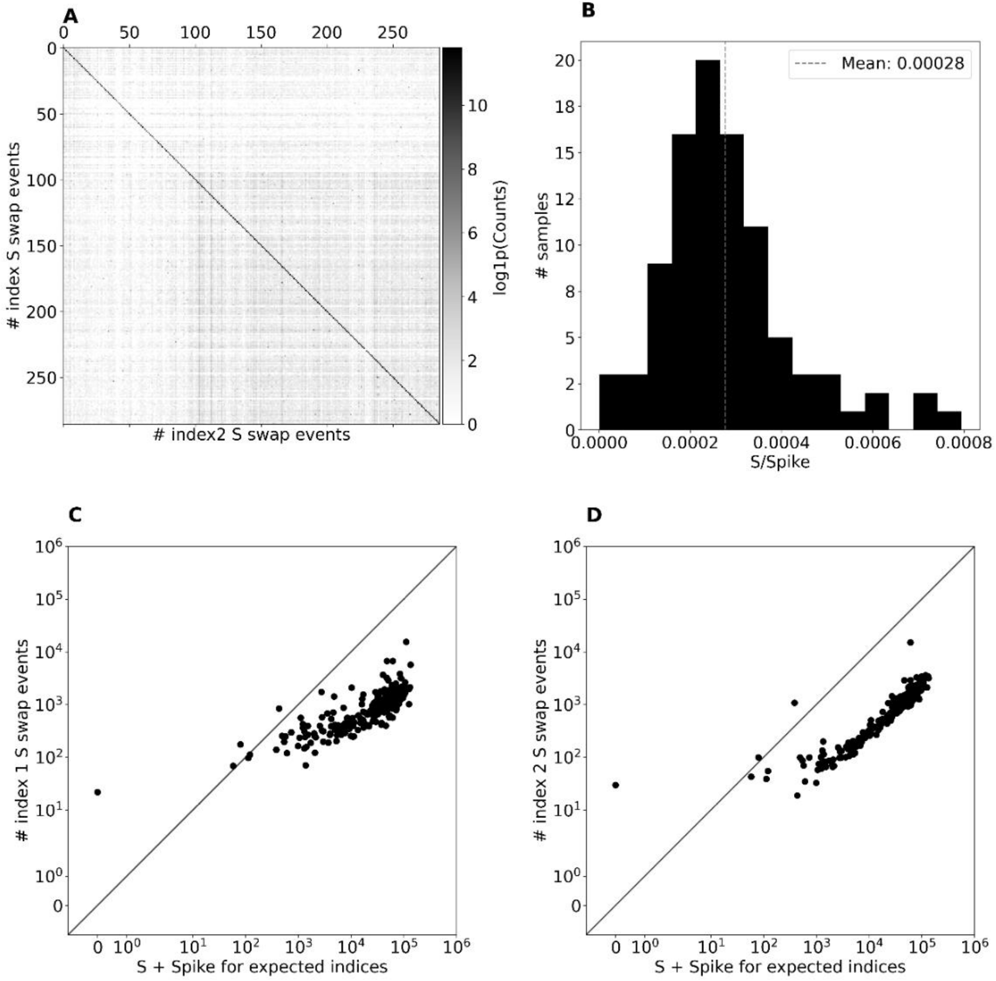

Figure S21. Quantifying the role of index mis-assignment as a source of noise in the S reads. A) A matching matrix for the viral S + S spike count for each pair of i5 and i7 index pairs from run v19 that used a unique dual index design. The index pairs along the diagonal correspond to expected index pairs for samples present in the experiment (expected matching indices) and the index pairs off of the diagonal correspond to index mis-assignment events. B) The distribution of ratios of viral S counts to Spike counts for samples with known zero amount of viral RNA. The mean ratio is 0.00028. C) The number of i7 mis-assignment events vs the number of viral S + S Spike counts for each sample. D) The number of i5 mis-assignment events vs the number of viral S + S Spike counts for each sample.

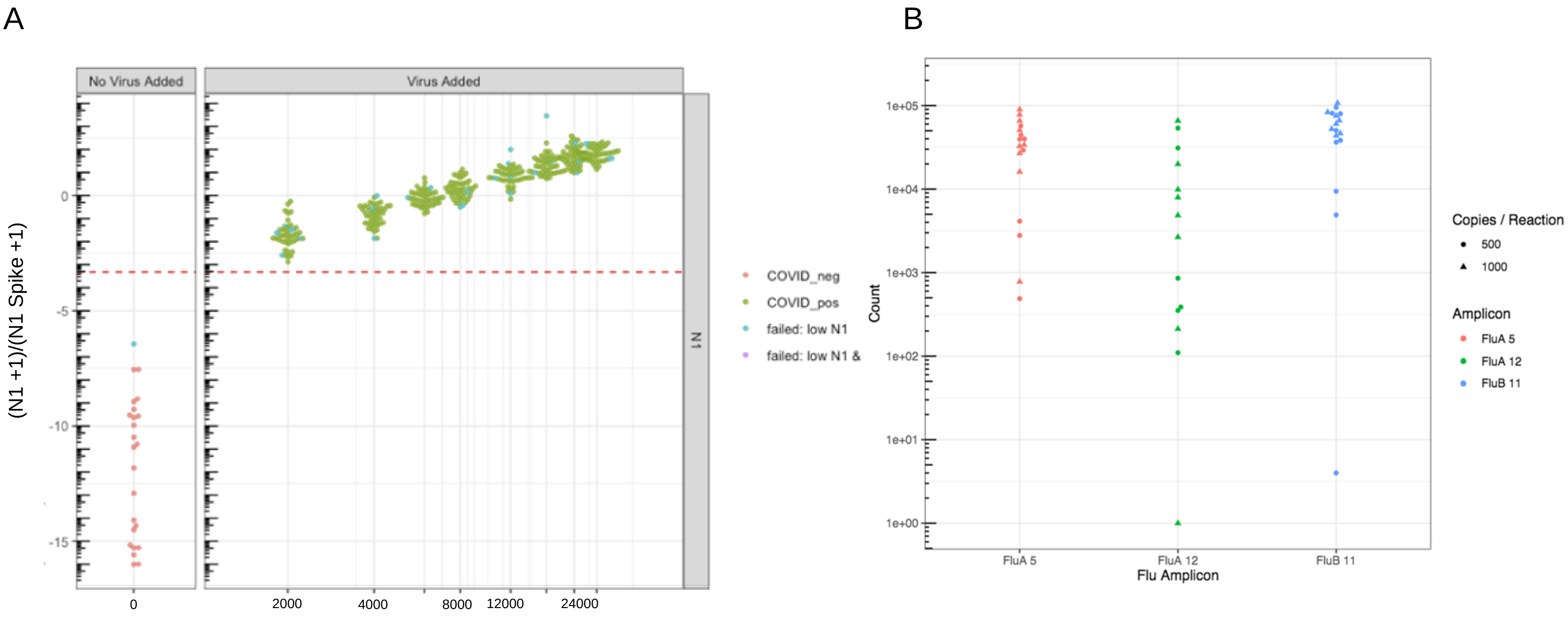

Figure S22. Extension of the SwabSeq Assay. A) We developed and tested to multiplex with additional SARS-CoV-2 amplicons. Here we demonstrate the N1 Amplicon LOD in saliva is around 2000 copies per mL. B) Testing of the three flu amplicons demonstrates that we are able to detect by sequencing samples with 500-1000 copies / reaction in PBS.
